# Antibody-dependent enhancement of severe dengue disease in humans

**DOI:** 10.1126/science.aan6836

**Published:** 2017-11-17

**Authors:** Leah C. Katzelnick, Lionel Gresh, M. Elizabeth Halloran, Juan Carlos Mercado, Guillermina Kuan, Aubree Gordon, Angel Balmaseda, Eva Harris

**Affiliations:** 1 Division of Infectious Diseases and Vaccinology, School of Public Health, University of California, Berkeley, CA, USA; 2 Sustainable Sciences Institute, Managua, Nicaragua; 3 Department of Biostatistics, University of Washington, WA, USA; 4 Vaccine and Infectious Disease Division, Fred Hutchinson Cancer Research Center, Seattle, WA, USA; 5 Laboratorio Nacional de Virología, Centro Nacional de Diagnóstico y Referencia, Ministry of Health, Managua, Nicaragua; 6 Centro de Salud Sócrates Flores Vivas, Ministry of Health, Managua, Nicaragua; 7 Department of Epidemiology, School of Public Health, University of Michigan, Ann Arbor, MI, USA

## Abstract

For dengue viruses 1 to 4 (DENV1-4), a specific range of antibody titer has been shown to enhance viral replication in vitro and severe disease in animal models. Although suspected, such antibody-dependent enhancement of severe disease has not been shown to occur in humans. Using multiple statistical approaches to study a long-term pediatric cohort in Nicaragua, we show that risk of severe dengue disease is highest within a narrow range of preexisting anti-DENV antibody titers. By contrast, we observe protection from all symptomatic dengue disease at high antibody titers. Thus, immune correlates of severe dengue must be evaluated separately from correlates of protection against symptomatic disease. These results have implications for studies of dengue pathogenesis and for vaccine development, because enhancement, not just lack of protection, is of concern.

Dengue viruses 1 to 4 (DENV1-4) are mosquitoborne flaviviruses that cause 50 to 100 million cases of dengue fever (DF) and ~500,000 hospitalizations annually (*1*, *2*). Dengue hemorrhagic fever/dengue shock syndrome (DHF/DSS) is the most severe form of dengue disease and is characterized by vascular leakage, hemorrhagic manifestations, thrombocytopenia, and hypotensive shock, which can lead to organ failure and death (*3*). Heterotypic secondary DENV infection (with a DENV type distinct from the primary infecting type) is the greatest risk factor for DHF/DSS (*4*, *5*). Age, interval between infections, antibody characteristics, viral factors, and host-specific genetics are contributing factors (*4*–*6*). The theory of antibody-dependent enhancement (ADE) posits that at a specific concentration, heterotypic antibodies bind but do not neutralize virions of the subsequent infecting DENV type. These virusimmune complexes are recognized by Fcg receptors that facilitate virus entry and replication in target immune cells. This initiates an immune cascade that results in vascular leak and severe dengue disease (*5*, *7*). In vitro and in animal models, a peak enhancement titer (i.e., a specific concentration of antibodies that most efficiently enhances DENV infection) has been observed. By contrast, higher antibody concentrations effectively neutralize virions, whereas lower concentrations poorly enhance infection (*8*, *9*).

However, there is no conclusive evidence in humans of a peak enhancement titer associated with the greatest risk of severe dengue disease. In a recent phase 3 clinical trial, young dengue vaccine recipients had elevated risk of dengue hospitalization >1 year after vaccination compared with placebo controls (*10*), raising concerns, but not confirming, that vaccination of DENV-naïve individuals induced poorly neutralizing anti-DENV antibodies that increased the risk of severe dengue disease (*11*). Further, the unexpected number of DHF/DSS cases in 6- to 12-month-old infants, when maternal derived–antibodies have decayed below neutralizing levels (*12*–*18*), is consistent with the concept of a peak enhancement titer for DHF/DSS. However, attempts to relate in vitro peak enhancement titer to disease severity in infants or older children have been inconclusive (*13*, *15*, *17*–*19*).

We directly studied the relationship between preexisting anti-DENV binding antibodies (DENV-Abs) and dengue disease severity in a large, well-characterized pediatric cohort study in Managua, Nicaragua (*20*, *21*). From August 2004 to April 2016, 8002 children aged 2 to 14 years were enrolled; 6684 children had at least one DENV-Ab titer measurement and were included in our study (41,302 samples in total) (fig. S1 and table S1). DENV-Ab titers were measured with the inhibition enzymelinked immunosorbent assay (iELISA) and estimated as the geometric mean of replicate titrations (quality control and reproducibility data in figs. S2 and S3) (*22*). In the iELISA, serially diluted serum antibodies compete with DENV-specific peroxidase-conjugated immunoglobulin G for binding to a balanced mixture of DENV1-4 antigens (*21*). The iELISA measures antibodies binding cross-reactive epitopes, such as the fusion loop in the envelope protein as well as the prM protein (table S2), that induce ADE in vitro and in vivo (*8*, *9*, *23*). For comparison, iELISA titers are reliably (Pearson’s correlation *r* = 80) a twofold dilution higher than hemagglutination inhibition assay titers (*24*) and are correlated {Pearson’s *r* = 0.80 [95% confidence interval (CI): 0.77 to 0.83]} with the geometric mean of neutralizing antibody titers to DENV1-4 (figs. S4 and S5 and table S3). As per the cohort protocol, children who became febrile visited the study health center, and those meeting the case definition for dengue or presenting with undifferentiated febrile illness were tested for dengue using molecular and serological diagnostic methods; those who developed warning signs for severe dengue disease were referred to the study hospital (618 dengue cases studied in total) (table S4) (*20*). Disease severity was initially classified using 1997 World Health Organization (WHO) criteria for DF and DHF/DSS (table S5) (*3*).

We first compared individuals with different levels of preexisting DENV-Ab titers (binned by fourfold serum dilution) to DENV-naïve individuals using a Cox proportional hazards model (*25*) adjusted for sex, epidemic season, age, and number of previous infections. Hazard ratio estimates of DHF/DSS across the range of DENV-Ab titers resembled the canonical ADE curve obtained in vitro (Figs. 1 and 2 and table S6) (*21*). The hazard of DHF/DSS was similar in children with no (DENV-naïve) or high (>1:1280) DENV-Ab titers. However, in children with preexisting DENV-Ab levels of 1:21 to 1:80, the hazard of DHF/DSS was 7.64-fold higher [95% CI: 3.19 to 18.28] (Fig. 1A). These effects remained significant when adjusted for age or number of previous infections (fig. S6) and when analyzed with alternative DENV-Ab titer binning methods or sampling of individual iELISA titer measurements (figs. S7 to S9). During the 12 years of the cohort studied, a child with preexisting DENV-Ab titers of 1:21 to 1:80 had a cumulative hazard of 11.4% for DHF/DSS. This is nearly twice as high as for a child with a prior DENV infection but low DENV-Ab titers (<1:21) who had a cumulative hazard of 6.6% of developing DHF/DSS (Fig. 1B). For DENV-naïve children and children with high DENV-Ab titers (>1:1280), the cumulative hazard was 1.6 and 1.5%, respectively, indicating that high antibody levels did not provide any greater protection against DHF/DSS than having no preexisting DENV-Abs. On average, in the Pediatric Dengue Cohort Study, the DENV-Ab half-life was 4.00 years [95% CI: 3.81 to 4.20], and by 3 years postinfection, an estimated 22% of children had DENV-Ab titers of 1:21 to 1:80 (table S7). Children with subsequent severe dengue cases had lower but not more rapidly decaying DENV-Ab titers (table S8).

**Fig. 1:**
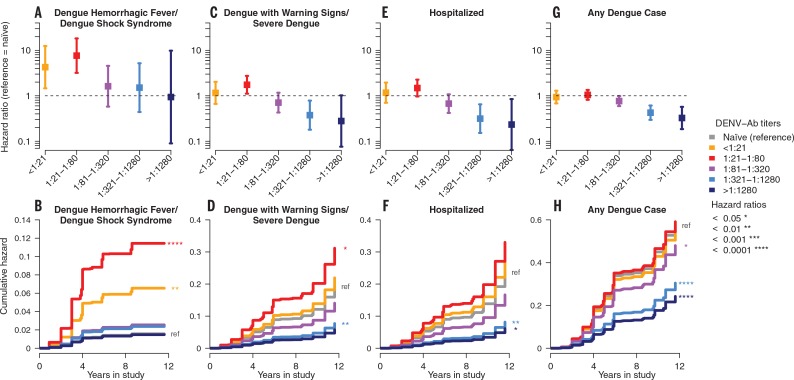
**Longitudinal analyses of the hazard of severe dengue disease or any dengue case by preexisting DENV-Ab titer for the full pediatric dengue cohort**. Hazard ratios with 95% CIs (**A, C, E**, and **G**) and cumulative hazard for an average child (**B, D, F**, and **H**) with preexisting DENV-Ab titers binned by fourfold dilution. Cox proportional hazard models were adjusted for sex, epidemic season, age, and number of previous DENV infections. Average child = female, age 5 to 9, 2007–2008 epidemic season, and one previous DENV infection.

**Fig. 2:**
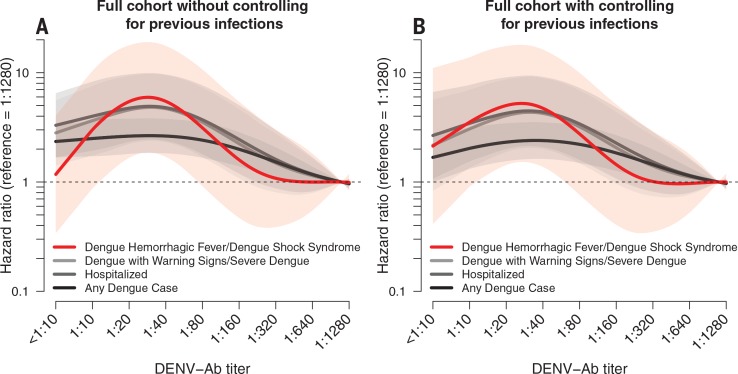
**Continuous hazard ratio curves for severe dengue disease or any dengue case by preexisting DENV-Ab titer for the pediatric dengue cohort**. Cox proportional hazard models were fit without (**A**) or with (**B**) control for number of previous infections. Models were also adjusted for sex, epidemic season, and age

In 2009, WHO revised the classification guidelines for severe dengue to improve clinical management of dengue patients and to capture other complications. The 2009 guidelines replace the category of DHF/DSS with “Dengue with Warning Signs” (Dengue+Warning Signs) and “Severe Dengue” (table S5) (*2*). We evaluated whether there is also a peak enhancement titer for Dengue+Warning Signs/Severe Dengue. Again, we observed that the highest hazard ratio, 1.75 [95% CI: 1.11 to 2.74], occurred among children with DENV-Ab titers of 1:21 to 1:80 (Fig. 1, C and D, and table S9). In children with higher antibody levels (1:321 to 1:1280 and >1:1280), the hazard ratios of Dengue+Warning Signs/Severe Dengue were less than those for DENV-naïve children, indicating that a protective effect against Dengue+Warning Signs/ Severe Dengue is also dependent on antibody titer. We also estimated hazard ratios for hospitalization admissions with dengue. Similarly, a peak enhancement titer was observed at DENV-Ab titers of 1:21 to 1:80, with protection observed at higher titers (Fig. 1, E and F, table S10; previous infections–only model, *P* < 0.05, fig. S6).

Hence, the magnitude of the observed enhancement effect related to how specific the definition of severe dengue disease was to the classical pathophysiological classification of DHF/DSS (*26*, *27*). When we relaxed the case definition criteria further and modeled the hazard of having any dengue case, we did not observe a peak enhancement titer: Children with a prior DENV infection and DENV-Ab titers <1:21 or 1:21 to 1:80 had comparable hazard ratios of dengue to DENV-naïve children (Fig. 1, G and H, and table S11). However, a protective effect was evident at DENV-Ab titers above 1:320.

Continuous hazard ratio curves for DHF/DSS rise and fall symmetrically around a peak hazard ratio of 5.95 [95% CI: 1.86 to 19.06], which occurred at a DENV-Ab titer of 1:34 (Fig. 2A) (*28*). When we controlled for prior DENV infection, children with DENV-Ab titers below this peak enhancement titer still had a lower hazard of DHF/DSS than those at the peak enhancement titer (Fig. 2B and fig. S10). Hazard ratio curves modeled on the basis of the WHO 2009 guidelines of Dengue+Warning Signs/Severe Dengue and hospitalization also peaked at 1:30, although the magnitude of the effect was smaller than for the DHF/DSS classification; again, protection was seen at higher DENV-Ab titers (Fig. 2, A and B).

To further test whether preexisting DENV-Ab levels explained our observations of a peak enhancement titer, we compared severe secondary dengue cases, each with five matched controls drawn randomly from the cohort. Controls were matched to cases by sex, age, and evidence of prior DENV infection but had not experienced dengue in the epidemic season of the case. Conditional logistic regression was used to compare the preexisting DENV-Ab titers of the severe cases with those of matched controls having titers >1:320. Again, a peak enhancement titer was observed at DENV-Ab titers of 1:21 to 1:80, with reduced odds ratios observed at lower and higher DENV-Ab titers (Fig. 3A and figs. S11 and S12), and the strongest enhancement effect was seen for the DHF/DSS classification (Fig. 3, A to C; titer distributions in Fig. 3, D to F). By contrast, when nonsevere secondary cases were compared to their own matched controls, no peak enhancement titer was observed (Fig. 3, A to C). We also observed the same peak enhancement titers when we directly compared severe to nonsevere dengue cases according to preinfection DENV-Ab titers, using logistic regression and accounting for known covariates (Fig. 4 and tables S12 to S14).

**Fig. 3:**
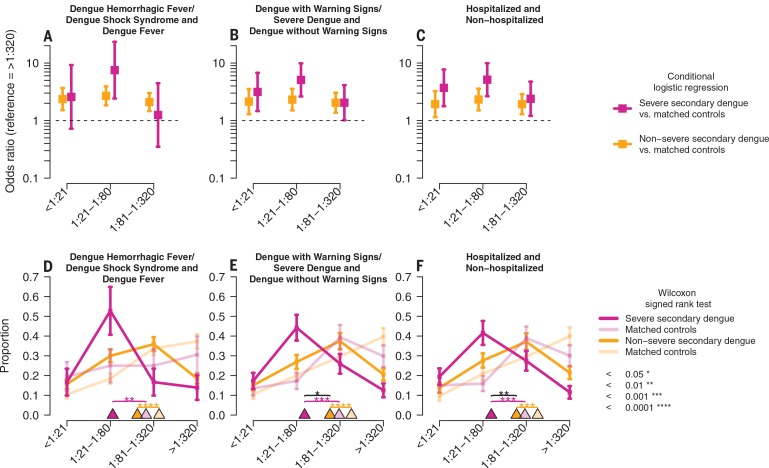
**Preexisting DENV-Ab titers in severe or nonsevere secondary dengue cases compared with matched controls drawn randomly from the pediatric dengue cohort.** (**A** to **C**) Five controls were matched to each case and were of the same sex and age, had evidence of prior DENV infection, provided a blood sample within 1 to 2 months of the case’s preinfection sample, but did not have a dengue case that year. Conditional logistic regression was used to compare preexisting DENV-Ab titers of severe cases and nonsevere cases each to matched controls, with titers >1:320 as reference. Odds ratios with 95% CIs are shown. (**D** to **F**) Distributions of preexisting DENV-Ab titers for severe and nonsevere secondary dengue cases and matched controls (one control for each case). Error bars show one SD, triangles show distribution medians, and brackets indicate significant differences in medians (severe and nonsevere cases compared with Wilcoxon rank sum test, black bracket).

**Fig. 4:**
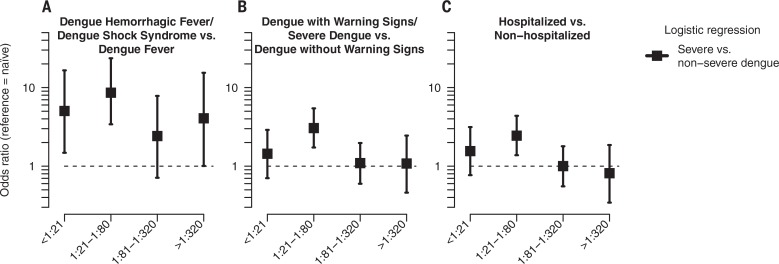
**Odds ratios for severe as compared with nonsevere dengue by preinfection DENV-Ab titer.** (**A** to **C**) Logistic regression models were adjusted for sex, epidemic season, infecting DENV type, age, and number of previous DENV infections. DENV-naïve children were used as the reference group. Odds ratios with 95% CIs are shown.

Thus, we have established that antibody titer does predict enhancement of severe dengue disease in humans. This association is seen most strongly for DHF/DSS, which is defined by the pathophysiological entity of DENV-induced vascular leak. By contrast, the 2009 WHO criteria, which are intentionally broader than DHF/DSS as they are meant improve triage and case management, encompass severe dengue cases caused by mechanisms other than vascular leak syndrome.

The correlate of risk for DHF/DSS and severe dengue disease (DENV-Ab titer 1:21 to 1:80) is distinct from the correlate of protection (DENVAb titers above 1:320) against symptomatic dengue. To date, dengue vaccine manufacturers have only been required to demonstrate induction of detectable neutralizing antibodies. With recent evidence of possible vaccine-enhanced dengue disease (*10*), this assumption is being revisited. A vaccine that induces antibody titers at or near the peak enhancement titer may place vaccinated individuals at greater risk of severe dengue than if they had never been vaccinated (*11*). Further, immune correlates of severe dengue need to be estimated separately from correlates of protection against any dengue disease in vaccine trials and natural infection studies, an observation that may also be relevant to studies of Zika disease and vaccines.

We show that the iELISA, a simpler assay than neutralization tests (*29*, *30*), is a tool for detection of elevated risk of severe disease as well as protection against symptomatic disease, making it a promising alternative method for measuring biologically predictive serological responses. Further, the iELISA measures antibodies targeting cross-reactive epitopes implicated in ADE in vitro and in vivo (*8*, *9*, *23*). The iELISA may directly measure the mechanistic correlate of enhancement and protection or may measure antibodies indirectly associated with the causal underlying immune determinants (*31*). Critical next steps toward identifying mechanistic correlates of protection and enhancement, as well as of safe, protective dengue vaccines, include development of serological assays that distinguish protective from enhancing antibodies; determination of how the sequence of infecting DENV types modifies disease; and integrated evaluation of cellular, innate, and humoral immunity to DENV infection and disease (*32*, *33*)*.*

In sum, we verify enhancement of dengue disease in humans and show that the level of preexisting anti-DENV antibodies is directly associated with the severity of secondary dengue disease in humans. We also show that the immune correlate for enhanced severe dengue disease is distinct from that for protection. These observations are important for future dengue and Zika vaccine trial design and evaluation, as well as for further studies on the mechanisms of ADE in relation to severe dengue and Zika disease.

## Supplementary Material

Antibody-dependent enhancement of severe dengue disease in humans

## SUPPLEMENTARY MATERIALS

www.sciencemag.org/content/358/6365/929/suppl/DC1

Tables S1 to S14

References (34–103)
