# Supplementary material for: Antibody-dependent enhancement of severe dengue disease in humans
Source: Science. 2017 Nov 17;358(6365):929–32. doi: 10.1126/science.aan6836 (PMC5858873; doi:10.1126/science.aan6836)
Supplement: Antibody-dependent enhancement of severe dengue disease in humans [file Science-358-929-s001.pdf]

www.sciencemag.org/cgi/content/full/science.aan6836/DC1

# Supplementary Materials for

## Antibody-dependent enhancement of severe dengue disease in humans

Leah C. Katzelnick, Lionel Gresh, M. Elizabeth Halloran, Juan Carlos Mercado, Guillermina Kuan, Aubree Gordon, Angel Balmaseda, Eva Harris*

*Corresponding author. Email: eharris@berkeley.edu

Published 2 November 2017 on *Science* First Release
DOI: 10.1126/science.aan6836

**This PDF file includes:**

Materials and Methods
Figs. S1 to S12
Tables S1 to S14
References

## Table of Contents


**Materials and Methods** **4**

Pediatric Dengue Cohort Study (PDCS). 4

Overview of annual sample collection and DENV-Ab measurement. 4

Background on the inhibition ELISA (iELISA). 4

DENV1-4 antigen production for the iELISA. 5

Conjugated competition antibody used for the iELISA. 6

Titration of DENV1-4 antigen and the conjugated competition antibody used in the iELISA. 6

Protocol for the iELISA (derived from the Standard Operating Procedure). 7

Quality control for the inhibition ELISA. 8

Reproducibility of the Inhibition ELISA. 9

Comparison of iELISA titers to hemagglutination inhibition assay titers. 9

Comparison of iELISA titers to neutralizing antibody titers from a flow-cytometry based assay. 9

Comparison of iELISA titers to percent neutralization in the Plaque Reduction Neutralization Test. 10

Background on DENV enhancing antibodies. 10

Comparison of iELISA titers to neutralization titers from children with known infection histories. 11

Characterization of DENV antigen used in the iELISA using a panel of mAbs. 11

Dengue case identification. 11

Severe vs. non-severe dengue definitions. 11

Immune history. 12

Inclusion criteria for dengue cases. 12

Longitudinal analyses of severe and symptomatic primary and secondary dengue. 12

Kinetics of DENV-Abs over time following DENV infection. 13

Nested case-control analyses for secondary severe and non-severe dengue. 13

Analyses of severe vs. non-severe dengue cases. 14

Potential implications of enhancement on transmission. 14

**Figures** **15**

Fig. S1. 15

Fig. S2. 16

Fig. S3. 18

Fig. S4. 19

Fig. S5. 20

Fig. S6. 21

Fig. S7. 22

Fig. S8. 24

Fig. S9. 25
Fig. S10. 26
Fig. S11. 27
Fig. S12. 28

**Tables 29**
Table S1. 29
Table S2. 30
Table S3. 31
Table S4. 32
Table S5. 33
Table S6. 34
Table. S7. 35
Table S8. 36
Table S9. 37
Table S10. 38
Table S11. 39
Table S12. 40
Table S13. 41
Table S14. 42

## Materials and Methods

Pediatric Dengue Cohort Study (PDCS).

The Nicaraguan PDCS (August 2004-present) has an active age-balanced cohort of ~3,500 children 2-14 years old. For this study, 6,684 cohort participants were included. Participants were recruited from neighborhoods served by the Health Center Sócrates Flores Vivas in District II of Managua, Nicaragua (*20*). Children were withdrawn when they turned 15, and new 2-year-old children were enrolled every year; additional children were recruited to balance the age distribution of the cohort. Parents or legal guardians of all subjects provided written informed consent, and subjects ≥6 years old provided assent (*20*). The PDCS was approved by the Institutional Review Boards of the Nicaraguan Ministry of Health, the University of California, Berkeley, and the University of Michigan.

Overview of annual sample collection and DENV-Ab measurement.

Each year, a healthy blood sample was collected from each participant. Anti-dengue virus binding antibody (DENV-Ab) titers were measured in paired annual samples (previous year and current year on the same plate) using an Inhibition ELISA (iELISA), in which serum or plasma antibodies were tested for their ability to block binding of DENV-specific peroxidase-conjugated IgG to a balanced mixture of DENV1-4 antigens (*34*) (described in detail below). Samples were tested for DENV-Abs by a single dilution (1:10) iELISA upon entry into the study or until DENV-Abs were observed. At that point, a serial dilution iELISA was performed on positive paired annual samples to estimate the 50% iELISA titer. For our analyses, iELISA titers derived from serial dilution always replaced single-dilution titers in our data set. The geometric mean of all serial dilution iELISA titers for a given sample (see section: "Reproducibility of the Inhibition ELISA") was used to reduce the effect of assay variability. Inapparent infections were defined as seroconversion (primary infection) or a ≥4-fold rise (secondary infection) in DENV-Ab titers between sequential annual samples. From August 2004 to April 2016, 8,002 children were enrolled in the PDCS and provided at least one blood sample to the cohort; 6,684 children had at least one DENV-Ab titer measurement at the time of analysis for this manuscript and were included in our study. The median number of annual samples each child contributed to the PDCS was 6 (IQR: 3-9). Approximately half of all samples had undetectable titers by iELISA (<1:10, 50.67%). Of those with detectable iELISA titers, the median titer was 1:201 (IQR: 1:60-1:514, distributions shown in in fig. S1A). Example trajectories of the iELISA for individual children are shown in fig. S1B.

Background on the inhibition ELISA (iELISA).

High-throughput, sensitive tools for measuring DENV-specific antibodies are needed for conducting seroprevalence surveys, confirming suspected DENV infections, and identifying inapparent DENV infections in longitudinal cohort studies. The traditional, gold-standard method for confirming DENV infections from paired acute-convalescent samples was the hemagglutination inhibition assay (HI) (*22*), but with the invention of enzyme-linked immunosorbent assays (ELISA), other methods were developed, including those directly measuring DENV-specific IgM and IgG antibodies. The advantage of the HI over assays that directly measure DENV-specific IgG by virtue of an OD reading is that it produces an antibody titer and measures 'functional' antibodies that prevent virion-mediated agglutination of red blood cells. The iELISA was developed as an alternative to the HI for DENV antibody measurement

and like the HI, also produces an antibody titer and measures how well antibodies in the test sample compete with antibodies known to strongly bind to DENV. The iELISA is easier than the HI in that primary chick or goose erythrocytes are not required, complex pH manipulations are not necessary, and the format is a standard ELISA format. Both the iELISA and the HI produce high cross-reactivity to all four DENV types and thus are not considered useful for identifying the previous or currently infecting DENV type. The plaque reduction neutralization test (PRNT, also focus reduction neutralization test, FRNT) is preferred for measuring neutralizing DENV antibodies and for confirming the infecting DENV type but is much more tedious, expensive, and time-consuming than either the HI or iELISA, and remains difficult to standardize (*35*). The iELISA is an ideal candidate for the high-throughput needs of many DENV cohort studies as well as Ministries of Health in low-resource countries, as it does not require primary erythrocytes, cell culture, or infectious virus, and most of the reagents can be made inexpensively in-house.

The iELISA was originally developed at the Instituto de Medicina Tropical "Pedro Kouri" in Havana, Cuba (*22*) (the technical manual can be found on the Pan American Health Organization website (*36*)). The iELISA, among other assays, is recommended for dengue case identification by the World Health Organization (*2*, *37*) as well as in prominent review papers (*38*). Further, the iELISA is more sensitive than the HI for identifying dengue cases and for primary/secondary case identification (*22*, *39*). The Nicaraguan Ministry of Health has used the iELISA for confirming primary and secondary dengue cases in research investigations since 1995, routine DENV case identification as part of the National Epidemiologic Dengue Surveillance Program, in the Nicaraguan Dengue Hospital Study since 1998, for seroprevalence estimation of a school-aged cohort from 2001-2003, and for serological diagnosis of cases and for annual seroprevalence and seroconversion measurement in the Pediatric Dengue Cohort Study since 2004 (*20*, *24*, *26*, *29*, *34*, *40–60*).

<u>DENV1-4 antigen production for the iELISA.</u>
As for the HI (*61*), iELISA antigen was prepared using the classical method of sucrose-acetone-treatment (*62*) of antigen derived from intracranial DENV inoculation of suckling mice, conducted according to the manual of the Instituto de Medicina Tropical "Pedro Kouri" (*36*). DENV prototype strains (DENV-1 Haw [Hawaii, 1944, Accession #: KM204119], DENV2 NGC [New Guinea, 1944, Accession #: KM204118], DENV3 H87 [Philippines, 1956, Accession #: KU050695], and DENV4 H241 [Philippines, 1956, Accession #: KR011349]) originated from the Center for Disease Control in Puerto Rico. Brain tissue was homogenized under cold conditions with 4 times the volume of tissue in distilled water with 8.5% sucrose. Cold acetone (10mL) was added to each antigen suspension in 8.5% sucrose, shaken vigorously, and left on ice for 15 minutes. The acetone was aspirated and removed, then replaced with 10mL of cold acetone and re-homogenized by shaking. The antigen suspension was stirred for 1 hour and allowed to sediment on ice for 30 minutes. Acetone was aspirated with a vacuum pump ($10^{-2}$mB) until the antigen suspension was completely dry (about 2 hours). The suspension was rehydrated with tris-borate-saline with 5% sucrose. For DENV4 antigen, half the volume of solution was added, and for DENV1, DENV2, and DENV3 antigen, the equivalent volume was added. The suspension was centrifuged for 30 minutes at 4°C at 3500 rpm. The supernatant containing the antigen was then aliquoted, inactivated for 40 minutes at 56°C, and stored at -70°C. The DENV type of each antigen was confirmed using specific primers for each DENV type (*63*) by RT-PCR. Equal volumes of the individually produced DENV1-4 antigen were mixed to produce the final

combined antigen. To confirm that equal amounts of the DENV1-4 antigens were present in the final antigen mixture, we used the antigen/conjugated antibody titration protocol (see section: "Titration of DENV1-4 antigen and the conjugated competition antibody used in the iELISA") to titrate each of the DENV1-4 antigens separately (1:10 starting dilution, four-fold serial dilutions to 1:640; a wider range of dilutions than the standard protocol was used to ensure detection of any differences) with different concentrations of the conjugated competition antibody (1:2000 – 1:8000). All antigens achieved similar optical density values at the same antigen and conjugated antibody dilutions.

Conjugated competition antibody used for the iELISA.

The conjugated competition antibody reagent was generated from a pool of de-identified human serum samples, each found to have iELISA titers of 1:10,000 to 1:100,000 during regular screening for DENV infections as part of the Nicaraguan Ministry of Health National Epidemiologic Dengue Surveillance program. The majority were convalescent samples (day 14-21 post-infection) from individuals who had symptomatic secondary DENV infections. All samples were collected before the introduction of Zika into Nicaragua in 2016. Only samples found to be IgM-negative by the IgM ELISA (*41*, *64*) were included in the pooled serum.

For antibody purification, 2mL of the high-iELISA titer serum pool was mixed with 2mL of PBS (pH 7.4) and stirred as 4mL of saturated ammonium sulfate solution was slowly added; the solution was stirred continuously for 45 minutes at room temperature, then centrifuged at 5000 rpm for 30 minutes at 4°C. The supernatant was discarded and the process repeated two more times. The final supernatant was re-suspended in 1mL PBS at pH 7.4, and the protein concentration was measured using a spectrophotometer at optical density of 260nm and 280nm. Using a molecular pore membrane, 1mL of the purified immunoglobulins at a concentration of 10mg/mL (diluted in 1X PBS) was dialyzed overnight in 1L carbonate-bicarbonate pH 9.5 at 4°C. Separately, 8mg of horseradish peroxidase VI fraction (5KU) was diluted in 1 mL of distilled water, added to 0.2mL sodium periodate (21mg/mL), and stirred slowly for 20 minutes at room temperature (covered to protect it from light). The peroxidase mixture was then dialyzed in 1L of sodium acetate (pH 4.4) at 4°C overnight. The dialyzed peroxidase solution was adjusted to pH 9.5 with carbonate-bicarbonate pH 9.5, mixed with the extracted antibody solution, and stirred at room temperature for 2 hours. Sodium borohydride (0.1 mL, 4mg/mL) was added to the solution and again stirred for 2 hours at 4°C. The conjugated antibody was precipitated with 2mL of saturated ammonium sulfate solution and stirred for 30 minutes at 4°C. The mixture was centrifuged at 3000 rpm for 30 minutes at 4°C, and the supernatant was discarded. The precipitate was resuspended in 0.3mL of PBS and dialyzed in 1L of 1X PBS at pH 4.5 for one day, with the PBS replaced three times. Following dialysis, 1% BSA and a 1:1 proportion of 99% glycerol was added to the conjugated antibody, and the stock was frozen at -20°C, protected from light.

Titration of DENV1-4 antigen and the conjugated competition antibody used in the iELISA.

Polystyrene plates (96 wells/plate) were treated with 100µL/well of anti-DENV polyclonal human IgG (in-house preparation derived from a pool of serum samples with high anti-DENV iELISA titer) at a protein concentration of 10µg/mL in carbonate-bicarbonate buffer at pH 9.6 (1.59 grams $Na_2CO_3$, 2.39 grams $NaHCO_3$, 1000 mL of distilled water). Plates were incubated overnight at ambient temperature in an incubator. Plates were then washed three times each with

280μL Phosphate Buffer Saline + 0.05% Tween 20 at pH 7.4 (PBS-T; 8.00g NaCl, 0.20g KCl, 0.14g $KH_2PO_4$, 0.91g $Na_2HPO_4$, 1000mL distilled water, 0.05% Tween 20). Wells were then blocked with 150μL of 1% Bovine Serum Albumin fraction V (BSA) in PBS-T and incubated 30 minutes at 37°C in an incubator. Plates were removed from the incubator, the blocking buffer removed, and 100μL/well was added of DENV1-4 antigen mixture diluted in PBS-T at serial dilutions from 1:50 to 1:200 of the initial antigen stock. Plates were incubated at 37°C for one hour and then washed four times with 280 μL/well PBS-T. The conjugated antibody was serially diluted from 1:2000 to 1:8000 in PBS-T with 1% normal human serum, and 100μL was added to each well, ensuring that for each antigen dilution there was a well of each conjugated competition antibody dilution. A negative control consisting of 1X PBS was added to wells with the serially diluted antigen to measure the level of background. Wells were incubated at 37°C for 30 minutes and washed four times with 280μL/well of PBS-T. Peroxidase substrate 3,3',5,5'-tetramethylbenzidine (TMB, Sigma-Alderich, 50μL/well) was added and incubated for 30 minutes at ambient room temperature, with plates covered to protect them from light. The reaction was stopped with 50μL of sulfuric acid, 2N solution, and plates were read on an ELISA reader at a wavelength of 450/630nm using ELISA Software version 1.09. The antigen titer and conjugated competition antibody titer used for the iELISA was the dilution at which the binding of conjugated antibody achieved an optical density (OD) value close to 1 and the greatest difference between the OD of the bound conjugated antibody and the PBS-only negative control (OD close to 0) was observed.

<u>Protocol for the iELISA (derived from the Standard Operating Procedure).</u>
The inhibition ELISA was performed as initially proposed by Fernández and Vázquez (*22*, *39*) with slight modifications. Anti-DENV polyclonal human IgG (10μg/mL) in carbonate-bicarbonate buffer at pH 9.6 (100μL/well) was added to 96-well polystyrene plates and incubated overnight at ambient temperature. Plates were washed three times with 280μL Phosphate Buffer Saline + 0.05% Tween 20 at pH 7.4 (PBS-T). Wells were blocked with 150μL of 1% BSA in PBS-T and incubated for 30 minutes at 37°C. Plates were removed from the incubator, the blocking buffer was removed, and 100μL/well was added of DENV1-4 antigen mixture diluted in PBS-T at the concentration determined in the antigen/conjugated competition antibody titration (see section: "Titration of DENV1-4 antigen and the conjugated competition antibody used in the iELISA"). Plates were incubated at 37°C for one hour, then washed four times with 280 μL/well of PBS-T. Serum samples were prepared either as a single 1:10 dilution (if the child was DENV-negative in the previous years) or a titration (if the child had been observed to have seroconverted in the past) consisting of ten-fold serial dilutions from a starting dilution of 1:10 to a dilution of 1:10,000 (for samples with iELISA titers found to be >1:10,000, the titration was repeated to include a 1:100,000 serum dilution). All samples were prepared in a total volume of 100μL/well of PBS-T with 0.5% BSA. A negative control consisting of normal human serum was added to four wells on the plate. The positive control consisted of high iELISA titer serum was diluted at a starting dilution of 1:100 to a dilution of 1:100,000 in four wells (see section on "Quality control of the iELISA", figs. S2, C to E). The plates were incubated for 2 hours at 37°C, then washed 4 times with 280μL/well of PBS-T. The conjugated antibody (100μL/well) was diluted in PBS-T with 1% normal human serum according to the titer obtained in the antigen/conjugated competition antibody titration protocol (see section: "Titration of DENV1-4 antigen and the conjugated competition antibodies used in the iELISA"). Plates were incubated at 37°C for 30 minutes, then washed four times with 280μL/well of PBS-T. Peroxidase substrate

TMB (50µL/well) was added, and plates were incubated for 30 minutes at ambient room temperature (covered to protect them from light). The reaction was stopped with 50µL of sulfuric acid, 2N solution. Plates were read on an ELISA reader at a wavelength of 450/630nm using ELISA Software version 1.09.

The mean of the absorbance values for negative controls was defined as 100% absorbance. The percent inhibition used for the single-dilution iELISA was calculated as 100*(1-[Sample absorbance/average negative control absorbance]). Paired samples (e.g., Year 1 and Year 2) in which Year 1 has <50% absorbance and Year 2 ≥50% absorbance at a 1:10 dilution were classified as a seroconversion. For samples in which <50% inhibition was achieved, the iELISA titer was reported as <1:10. For participants who had seroconverted in the past, a serum titration of paired annual samples was used to estimate iELISA titers (fig. S2, A and B). Percent absorbance for the serum sample dilutions was measured relative to the negative control using the Reed-Muench method (*65*):

Eq. $10\text{^}(\log_{10}[T_{\geq 50\%}] + [D_{\geq 50\%} - 50]/[D_{\geq 50\%} - D_{<50\%}])$

where $D_{\geq 50\%}$ is the percent inhibition of the last serum dilution with ≥50% inhibition, $T_{\geq 50\%}$ is the last serum dilution at which ≥50% inhibition is observed, and $D_{<50\%}$ is the percent inhibition of the first serum dilution with <50% inhibition.

For example, a child with 70% inhibition at a 1:10 serum dilution and 20% inhibition at a 1:100 serum dilution would have an iELISA titer of $10\text{^}(\log_{10}[10] + [70 - 50]/[70 - 20]) = 25.11886$, which would be rounded to 25 and reported as 1:25.

Quality control for the inhibition ELISA.

Proficiency panels provided by the Instituto de Medicina Tropical "Pedro Kouri" were used to assess the performance of the iELISA. Further, on each plate and for each time the iELISA was run, a positive control and negative control were included. The negative control was composed of DENV-naïve normal human serum and was run in quadruplicate. The mean of the absorbance values for negative controls had to have an optical density ≥0.8 and ≤1.5 for the experiment to be valid; data were discarded and samples repeated if values outside this range were observed (fig. S2C). The positive control was from an individual with an iELISA titer of >1:10,000 identified through screening for dengue as part of the Nicaraguan Ministry of Health National Epidemiologic Dengue Surveillance program. The positive control was tested in each experiment at dilutions of 1:100 to 1:100,000 (fig. S2D), and the measured iELISA titer in each experiment had to be >10,000 or else the results were not deemed valid and the data were discarded. Multiple times per year, eight antisera with established iELISA titers were titrated to ensure that the assay was producing consistent values (within two-fold of the established titer). We tested for systematic deviations in iELISA titers by measuring the difference between individual iELISA titers and the geometric mean titer (GMT) for that sample according to day of titration. We found that deviations in iELISA titers by day of titration were rarely more than two-fold (fig. S2E); further, we found no evidence for systematic differences over the period of the cohort (slope = -0.00042 [95%CI:-0.0010-0.00019]).

Reproducibility of the Inhibition ELISA.

In the PDCS, DENV-specific seroconversion is measured each year using paired annual samples; thus, the majority of samples are titrated at least twice, and on different days, by the iELISA. Additionally, each year, a random 20% of paired annual samples are tested twice. All children are screened by single-dilution iELISA titer upon entry into the cohort study and until they are observed to be positive by single-dilution iELISA. In total, 31,341 samples were tested by single-dilution iELISA, and half (n=19,856) did not have detectable DENV-Abs. Once a child seroconverted, iELISA titrations (1:10 to 1:10,000) were conducted on the seroconverted sample and on all subsequent samples (n=22,373). The majority of these samples (66%) were titrated two or more times by iELISA on different days (fig. S3A). Differences between repeat iELISA titrations were mostly (65%) <2-fold; 87% differed by <4-fold and 95% by <8-fold (fig. S3B). The vast majority (75-93%, depending on the bin) of individual iELISA titers fell within the same bin as the average iELISA titer for that sample (fig. S3C).

Comparison of iELISA titers to hemagglutination inhibition assay titers.

The HI assay is a classic assay traditionally used in the flavivirus field for measuring total anti-DENV antibody responses. In this assay, test sera are serially diluted and each dilution is evaluated to determine its ability to inhibit agglutination of goose red blood cells. It was established decades before what we now know about the structure and biology of flaviviruses. When we initiated our clinical and epidemiological studies in Nicaragua in 1998, the HI assay was considered a standard and accepted serological method in the flavivirus field, and thus was used as a benchmark for the iELISA, which is a simplified version that measures the ability of serial dilutions of test sera to inhibit binding to DENV antigen. The HI is used for measuring serological responses in multiple cohort studies in Asia (*61*, *66*). In a school-aged cohort conducted in Managua, Nicaragua from 2001-2003, the iELISA measurement of seropositivity was compared to seropositivity measured by the HI using the Clarke and Casals method (*62*) for 197 samples (21.2% of all samples collected), yielding a sensitivity of 98.9% and specificity of 100%. Antibody titers as measured by the iELISA and HI were also compared for 106 samples (11.4% of the study population), resulting in a Pearson's correlation of 0.80. HI titers were found to be a two-fold dilution lower than iELISA titers (*24*).

Comparison of iELISA titers to neutralizing antibody titers from a flow-cytometry based assay.

A subset of children from the PDCS (n=112) was selected for in-depth serological analysis with a flow-cytometry based neutralization assay based on infection of Raji-DC-SIGN cells with reporter virus particles (RVPs) of DENV1-4 (DENV1 Western Pacific 74, Accession # = AY145121; DENV2 S16803, Accession # = GU289914; DENV3 CH53489, Accession # = DQ863638; and DENV4 TVP360, Accession # = KU513442), as described previously (*29*, *43*). Neutralizing antibodies were measured in annual serum samples from 18 children with one DENV infection, 62 children with two DENV infections, and 32 children with three DENV infections. Neutralizing antibody titers ($NT_{50}$) were measured as the reciprocal serum dilution in which 50% of viruses were neutralized relative to wells in which no serum is present.

We compared iELISA titers to the $NT_{50}$ titers for each DENV type, as well as the mean and median of the $NT_{50}$ titers against the DENV1-4 for each sample. We use the leiv package in R (Bivariate Linear Errors-in-Variables Estimator (*67*)) to estimate the relationship between iELISA titers and $NT_{50}$ titers, allowing for measurement error in both variables. There was a strong relationship between iELISA titers and $NT_{50}$ titers to each individual DENV type (fig. S4,

table S3A), with slopes close to 1 for DENV1, DENV2, and DENV3. The strongest correlation was between iELISA titers and the mean of the four $NT_{50}$ titers for any sample (Pearson's correlation of 0.803 [95%CI: 0.776 - 0.827]); iELISA titers were also strongly correlated with the median of $NT_{50}$ titers to DENV1-4 (0.796 [95%CI: 0.768 - 0.82]).

Comparison of iELISA titers to percent neutralization in the Plaque Reduction Neutralization Test.

In some cohort studies, a single-dilution or two-dilution PRNT is conducted to estimate neutralization against each DENV type (*4*, *68*, *69*). Two data sets from our previous studies were available for analyzing the relationship between the iELISA and the single-dilution PRNT and were pooled for this analysis. First, a random 10% of PDCS participants (n=371) from 2004-2007 were tested for neutralizing antibodies using a single-dilution plaque reduction neutralization test (1:30 serum-dilution, n=1484 samples). An additional 299 cohort participants who experienced a DENV infection based on annual iELISA seroconversion in 2004-2008 had percent PRNT reduction against DENV1-4 measured before and after their purported infection (total of 598 paired annual samples). The PRNT was conducted with BHK21 cells in 12-well plates using reference strains for DENV1-4 (DENV1 West Pac, DENV2 16681, DENV3 3009, DENV4 TVP 360). After pre-incubation of the serum-virus mixture for 1 hour at 37°C (150µL serum, 150µL containing 40 pfu virus), the serum-virus mixture was added to cells to allow infection for 1 hour at 37°C. An overlay medium with low melting-point (LMP) agar and 2X MEM was added, and plaques were allowed to grow for 5-7 days at 37°C. Plates were fixed with 10% formaldehyde, and plaques were visualized with crystal violet. As we found for the $NT_{50}$ titers obtained using the flow-cytometry assay, means and medians of percent PRNT reductions were strongly correlated to iELISA titers (Pearson's r=0.727 [0.706 - 0.747]) (table S3B).

Background on DENV enhancing antibodies.

Most neutralizing anti-DENV monoclonal antibodies (mAbs) at sub-neutralizing concentrations are capable of enhancing DENV *in vitro* (*70*), but the extent to which they enhance varies greatly. The majority of human mAbs derived from memory B cells after primary DENV infection are subclass IgG1 and target cross-reactive epitopes, specifically the fusion loop on DII of the E protein (*71*) and the pr portion of the prM/M protein (*70*, *72*, *73*). The enhancing component of the cross-reactive serum antibody repertoire is also dominated by anti-E fusion loop and anti-prM antibodies that target distinct epitopes and thus both anti-fusion loop and anti-prM antibodies may independently contribute to ADE (*23*). Antibodies directed to the E fusion loop are varied; some can potently neutralize *in vitro*, and in general these antibodies have a wide range of concentrations over which they enhance both mature and immature DENV infection of K562 cells (which contain FcγRIIa) (*70*). Anti-prM antibodies poorly neutralize even partially immature virus and most also enhance over a wide range of concentrations (*72*). During infection, human DENV virions are thought to be mostly mature (DENV grown in human B cells are mostly mature (*72*)), and thus the extent to which prM antibodies are relevant to ADE in human infections remains an unanswered question. The composition of the Fc portion of antibodies is also likely important for ADE. The majority of anti-DENV antibodies are IgG1 and IgG3, which both bind FcγRIIa. Host polymorphisms that modify affinity for FcγRIIa have been associated with symptomatic dengue and ADE of infection (*6*, *74–76*), and differences in antibody affinity to FcγRIIIa have also been associated with disease severity (*6*).

Comparison of iELISA titers to neutralization titers from children with known infection histories.

We also compared iELISA titers to neutralization titers ($NT_{50}$) to DENV1-4 in samples from children with previous primary DENV1 or DENV2 infections (fig. S5, A and B; not enough primary DENV3 or DENV4 infections were available for analysis). For children with primary DENV1 infections, $NT_{50}$ titers to DENV1-4 were strongly related to iELISA titers (slopes 0.8-1.51), but DENV1 $NT_{50}$ titers were consistently higher than iELISA titers, while DENV2 and DENV3 $NT_{50}$ titers were about the same as the iELISA, and DENV4 titers were lower. A similar scenario was observed with primary DENV2 responses: the $NT_{50}$ to DENV2 was systematically higher than the iELISA titers, while the $NT_{50}$ to DENV1, DENV3, and DENV4 were about the same or slightly lower than the iELISA titers. That the strongest correspondence (i.e. closest to slope=1, intercept=0) was between iELISA titers and $NT_{50}$ titers to heterotypic DENV types suggests that the iELISA is most related to cross-reactive neutralizing responses following primary DENV infection.

Characterization of DENV antigen used in the iELISA using a panel of mAbs.

To characterize the epitopes recognized using the iELISA with sucrose-acetone treated antigen, a panel of human and mouse mAbs targeting type-specific and cross-reactive enhancing epitopes were titrated in the iELISA at high concentrations (dilutions: 0.1 mg/mL, 0.01 mg/mL, 0.001 mg/mL, 0.0001 mg/mL) in two independent experiments (table S2). Even at mAb concentrations of 0.1 mg/mL and 0.01 mg/mL, only a subset of the mAbs was inhibitory. This included mAbs 4G2 (fusion loop (*77*)) and 2H2 (prM (*78*)), which target epitopes that are primarily associated with ADE *in vitro* and *in vivo*; antibodies targeting potently neutralizing quaternary epitopes (both type-specific and cross-reactive, including anti-DENV1 1F4 (*79*), anti-DENV2 2D22 (*80*), anti-DENV3 5J7 (*81*), cross-reactive EDE (*82*)) were not inhibitory. MAbs 1C19 (BC loop, adjacent to fusion loop (*83*)), 1M7 (fusion loop (*83*)), DV2-87 (EDIII CC loop (*84*)), and E1D8 (NS3 (*85*)) were also inhibitory. Based on these observations, we believe the iELISA primarily measures antibodies binding to cross-reactive epitopes, some of which are associated with enhancement *in vitro*.

Dengue case identification.

Children in the PDCS who became febrile visited the Health Center Sócrates Flores Vivas and were clinically screened for dengue using the WHO case definition. Acute and convalescent blood samples were collected from children with suspected dengue or undifferentiated febrile illnesses. Paired samples with seroconversion by IgM ELISA, and/or seroconversion, and/or a $\geq$4-fold rise in DENV-Abs by IE, and/or acute samples positive for DENV by RT-PCR, and/or virus isolation were documented as symptomatic dengue cases.

Severe vs. non-severe dengue definitions.

Children were monitored throughout their illness, and those who developed signs of severe dengue disease were admitted to the study hospital, the National Pediatric Reference Hospital in Managua. Disease severity was classified using: the 1997 WHO criteria (DF, DHF, and DSS) (*3*); the revised 2009 WHO criteria ("Dengue without Warning Signs", "Dengue with Warning Signs", and "Severe Dengue") (*2*); and a clinical management score to define severe disease (tables S4 and S5) (*26*, *27*). In the latter, Category I cases were treated in the outpatient clinic; Category II cases were hospitalized and given intravenous fluids for rehydration; and Category

III cases were hospitalized and/or treated in the intensive care unit (*26*). Due to low numbers, we analyzed the outcomes as 'severe dengue' and 'non-severe dengue' for each criterion: DHF/DSS vs. DF (1997 WHO Criteria), Dengue+Warning Signs/Severe Dengue vs. Dengue without Warning Signs (2009 WHO criteria), and Hospitalized (Category II/III) vs. Non-hospitalized (Category I) (clinical management score).

<u>Immune history.</u>
Dengue cases were defined as secondary infections if the child had a previous documented symptomatic infection or seroconverted or had an iELISA titer >1:10 at any point in the years leading up to his/her dengue case. Dengue cases were identified as primary infections if the child had no evidence of prior DENV infection or immunity as measured in annual samples for all years prior to the dengue case. Some children entered the cohort DENV-immune and thus it is not known how many previous infections they have had.

<u>Inclusion criteria for dengue cases.</u>
Between August 30, 2004 and April 1, 2016, there were 645 dengue cases. Twenty-four cases did not have a DENV-Ab titer measured within the previous year, and three cases had symptomatic infections but no information on disease severity; these were excluded. In total, 618 cases (295 primary and 323 secondary) were included in our analyses. The number of children with one, two or three observed dengue cases was 573, 21, and one, respectively.

<u>Longitudinal analyses of severe and symptomatic primary and secondary dengue.</u>
All analyses were conducted with R 3.3.2 (R Foundation for Statistical Computing). Cox proportional hazards models (R survival package (*25*)) were used to estimate the hazard ratios and cumulative hazard of severe or symptomatic dengue in the PDCS by pre-existing DENV-Ab titer. Sex, epidemic season, age (2-4, 5-9, 10-14) and number of previous infections (0, 1, ≥2) were included as covariates (covariate coefficients in tables S6, S9 to S11). DENV-Ab titer was analyzed as a time-dependent covariate constant between DENV-Ab titer measurements or from the last titer measurement to exit from the cohort. DENV-Ab titers were binned by four-fold dilution in the iELISA assay (<1:21, 1:21-1:80, 1:81-1:320, 1:321-1:1280, >1:1280), with DENV-naïve-children as the reference group. We estimated a cluster variance to control for correlation within subjects. Sensitivity analyses include: models controlling for either age or number of previous infections (fig. S6) and models in which DENV-Ab titer were binned by quartiles, quintiles, or sextiles of all DENV-Ab titers for non-naïve children in the PDCS (fig. S7). To evaluate whether model results would have differed if any individual iELISA titer, rather than the geometric mean titer (GMT), were selected for analyses, we randomly sampled one of the repeat iELISA titrations for each sample to create 50 data sets made up of different iELISA titrations each time to estimate hazard ratios for severe and symptomatic dengue. Overall, estimates with randomly sampled iELISA titers as predictors (fig. S8) were similar to those acquired with GMTs (Fig. 1), including statistically significant enhancement of DHF/DSS at DENV-Ab titers of 1:21-1:80 and protection against symptomatic dengue at high (>1:80) DENV-Ab titers.

We used the particular epidemiology of Nicaragua to test whether pre-infection DENV-Ab titers in a population not previously exposed to DENV3 predicted disease outcome during the re-introduction of DENV3 into Nicaragua in 2008-2011 after an absence (no virologically

confirmed cases) of DENV3 since 1998 (*51*). We excluded children who had documented evidence of two or more infections as well as any samples taken after the child experienced a DENV infection (inapparent or symptomatic) between 2008-2011. Simple logistic regression was used to estimate odds ratios of dengue disease severity by iELISA titers. Although the sample size is relatively small, we find similar trends to those for hazard ratio estimates for the full cohort, including elevated hazard ratios for DHF/DSS at titers of 1:21-1:80 (fig. S9).

We also created spline-based hazard ratio curves for DENV-Ab titers analyzed as a continuous predictor (smoothHR package (*28*)), using high DENV-Ab titers (1:1280) as the reference. We created separate models that included all children and were adjusted for sex, epidemic season, age and with and without adjustment for number of previous infections (Fig. 2) as well as a model that included only non-naïve children and was adjusted for sex, epidemic season, age and number of previous infections (fig. S10). We also identified the iELISA titer corresponding to the highest point on the predicted hazard ratio curve and expressed this as the peak enhancement titer.

Kinetics of DENV-Abs over time following DENV infection.

We estimated the rate of antibody decay over time between DENV infections according to the disease severity of the subsequent DENV infection. We used a linear mixed-effects model (lme4 and merTools packages in R (*86*, *87*)), controlling for number of previous infections, with random effects to account for differences in individual intercepts and slopes, and an interaction term for antibody decay and subsequent disease severity. We selected this model based on previous research suggesting that linear models of logged antibody titers are sufficient for fitting long-term immune responses (>4 months), consistent with the period of time between most DENV infections and annual serum sampling in the PDCS (*88*). We found that children who developed dengue disease had lower DENV-Ab titers ($\log_2$ -0.89 [95%CI: -1.71--0.08] lower for DHF/DSS, table S8) immediately after their infection than those who did not. However, we did not observe significant differences in decay by subsequent severe disease ($\log_2$ -0.01 [-0.15-0.14] for DHF/DSS, table S8), suggesting that DENV-Ab titers do not decay more quickly in those who subsequently develop severe dengue. We also used simple linear models to estimate the slopes and intercepts for each child separately, and obtained similar results: intercepts differed significantly between those who did and did not develop severe dengue ($p<0.05$), but decay rates did not ($p>0.05$).

We also estimated rates of antibody decay in the cohort as well as the proportion of children expected to have DENV-Ab titers of 1:21-1:80 (enhancement window) or >1:80 (protective range) by the number of years after their previous infection (table S7). We observe an antibody half-life ($1/_{\log 2}$ [decay rate]) of 4.00 years [95%CI: 3.81-4.20]. By three years post-infection, 71% of children had DENV-Ab titers in the protective range while 22% had DENV-Ab titers within the enhancing window.

Nested case-control analyses for secondary severe and non-severe dengue.

A nested case-control design was used to evaluate whether pre-existing DENV-Ab titers were associated with odds of being a severe or non-severe secondary dengue case. Controls (5 controls per case, distributions shown in fig. S11) had evidence of prior DENV infection and were matched to cases by birth year (within one year of the birthdate of the case), sex, and year of sampling (samples drawn within 1-2 months of the sample for the case) but either did not have a

dengue case that year (Fig. 3) or did not have a dengue case during the cohort (fig. S12). We used conditional logistic regression (R survival package (*25*)) to compare pre-existing DENV-Ab titers binned by four-fold dilution in the iELISA assay (<1:21, 1:21-1:80, 1:81-1:320, >1:320, with >1:320 as reference group—low numbers precluded analyzing 1:321-1:1280 and >1:1280 groups separately) between non-severe or severe secondary dengue cases and their matched controls. We also estimated distributions of DENV-Ab titers for these groups and tested for significant differences in medians with the Wilcoxon signed rank test (1 control/case) with a continuity correction. Wilcoxon rank sum test was used to compare severe and non-severe secondary dengue cases.

Analyses of severe vs. non-severe dengue cases.

Logistic regression was used to estimate the odds ratios of severe as compared to non-severe dengue (analyzed separately for each disease severity classification scheme) controlling for sex, epidemic season, DENV type, age, and number of previous infections (tables S12 to S14). Pre-existing DENV-Ab titers were binned by four-fold dilution in the iELISA assay (<1:21, 1:21-1:80, 1:81-1:320, and >1:320), with DENV-naïve children as the reference group.

Potential implications of enhancement on transmission.

The potential effect of antibody-dependent enhancement on DENV transmission has been explored with multiple theoretical and statistical models, with some showing that it could help explain the dynamics of the four DENV types (*89*, *90*) and others finding that enhancement was not necessary to explain these dynamics (*91*, *92*). Based on empirical data, peak viremia is significantly higher in children with DHF than DF, and viral clearance is more rapid in secondary dengue cases (*93–95*). Within-host models of these time-resolved viremia measurements have been used to test competing hypotheses of the immunological mechanisms that control viremia levels and differences between severe and non-severe patients, observing that models incorporating ADE as well as T-cell mediated protection are consistent with observed viral kinetics (*96*, *97*). A study measuring transmission to *Aedes aegypti* mosquitoes observed that the magnitude of viremia correlates with the proportion of infected mosquitoes, duration of transmissibility to the mosquito, and proportion of mosquitoes with infectious saliva at day 14 post-feeding (*98*). Others have found that at a given viremia level, asymptomatic individuals may be more infectious than symptomatic individuals, but symptomatic dengue cases overall have higher viremia (*99*). A better understanding of the relationship between duration and magnitude of viremia and overall transmission probability is critical for building more accurate and predictive DENV transmission models (*100*, *101*). Index-cluster studies provide a direct way to measure the relationship between disease and probability of transmission (*49*, *102*); however, to our knowledge, no studies have explicitly measured transmissibility in severe versus non-severe dengue cases.

## Figures

**Fig. S1.**

Inhibition ELISA (iELISA) titers in the Pediatric Dengue Cohort Study (PDCS). (**A**) Number of samples in each iELISA two-fold titer bin for all 12 years of the PDCS studied. **(B)** Examples of iELISA titer trajectories over the course of the PDCS for a random selection of children (sampled from those with ≥5 annual samples collected to enable visualization of trajectories).

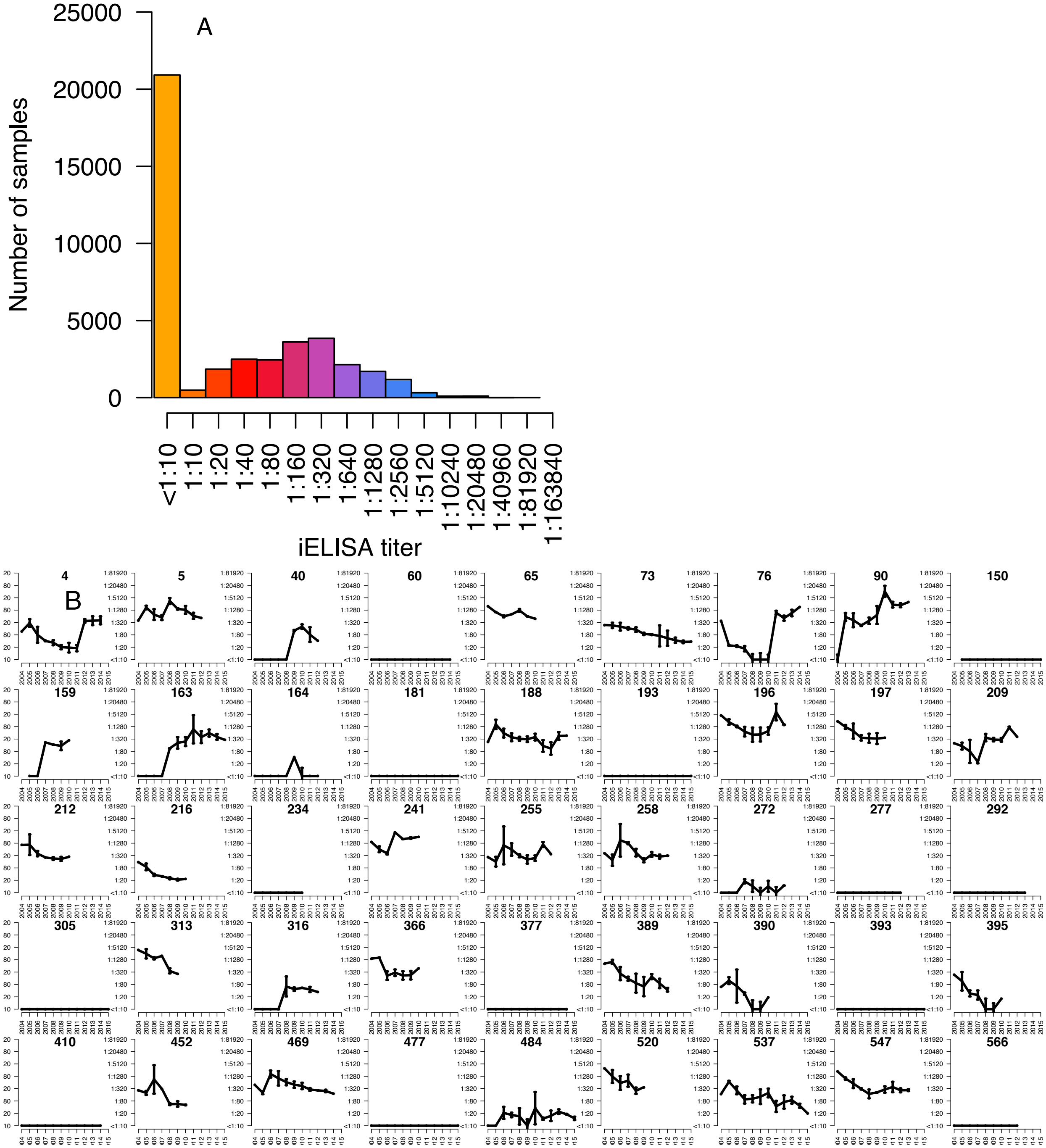

**Fig. S2.**

Raw iELISA titration data and iELISA quality control. (**A**) Representative optical density (OD) data from iELISA titrations for paired annual samples (Year-1 and Year-2) for two children (S1, previously immune, no infection between Year-1 and Year-2; and S2, seroconversion between Year-1 and Year-2). **(B)** Corresponding percent inhibition estimated from the optical density data in **A**, with estimated iELISA titers. (**C-D**) Ranges of the negative (**C**) and positive (**D**) controls over a period of time at the National Virology Laboratory, National Center of Diagnostics and Reference, Nicaraguan Ministry of Health. (**E**) Boxplots showing fold-deviation in individual iELISA titers from the geometric mean titer for that sample by day of titration. The red line is a linear regression of the median fold-deviations by day of titration (slope = 0.00042 [95%CI:-0.0010-0.00019]).

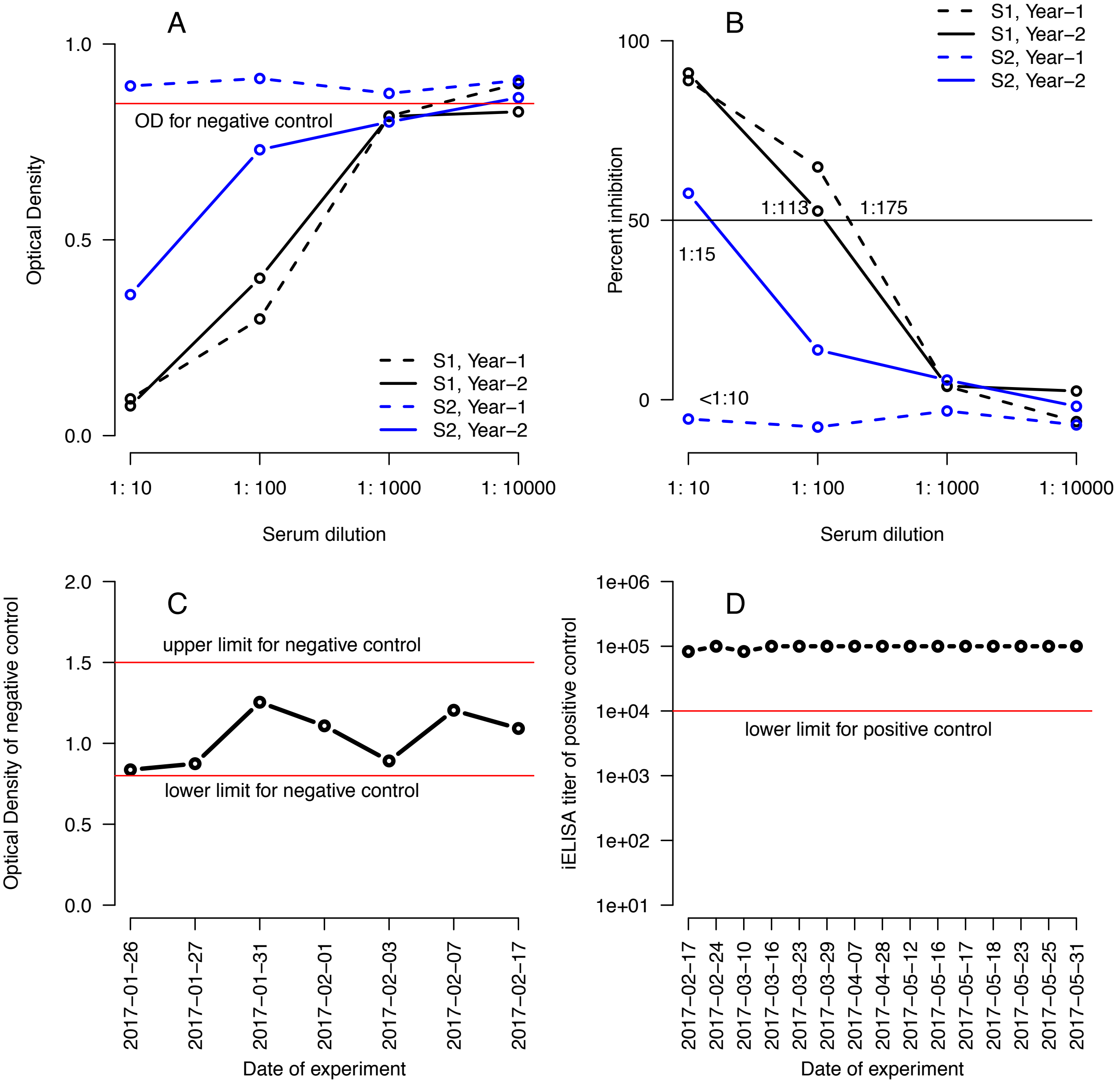

**Fig. S2 continued.**

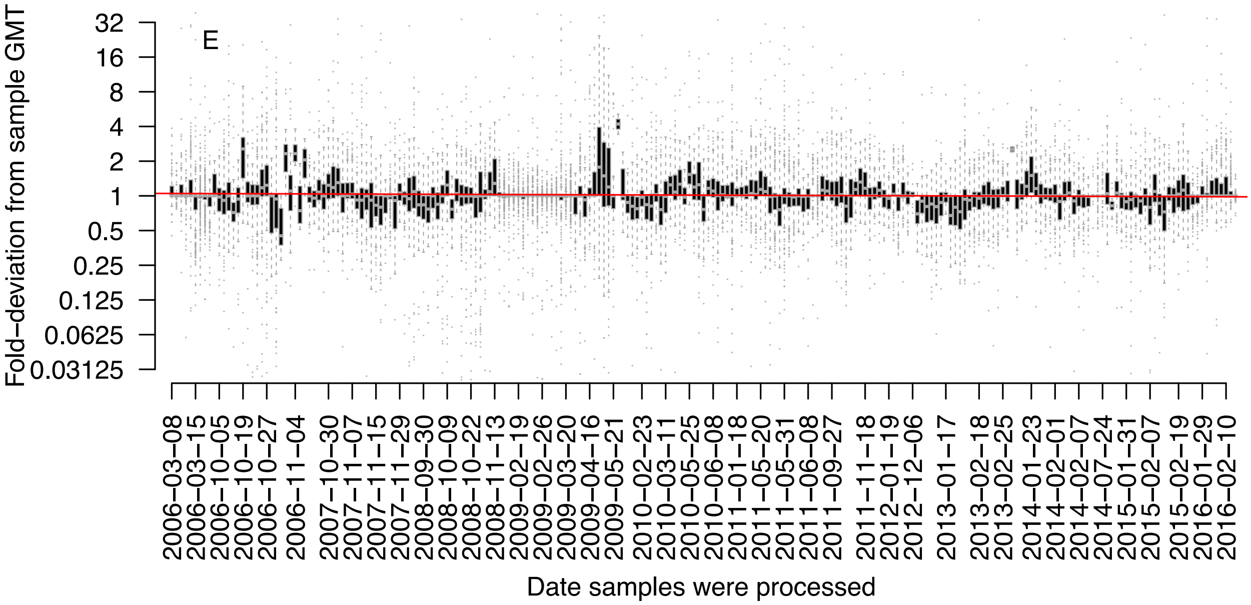

**Fig. S3.**

Reproducibility of the iELISA. (**A**) Distribution of the number of repeat iELISA titrations for each sample. Singlets often occur in the year when children enter the cohort or in the year before they leave the cohort, as the sample is only tested once in paired annual samples. (**B**) Distribution of fold-differences in iELISA repeats. Percentages indicate proportion of samples with iELISA titers less than the fold-difference indicated on the x-axis. (**C**) Distributions of iELISA titers by the bin of the GMT for that sample. Percentages indicate the number of individual iELISA titers that fall into the same bin as the GMT for that sample.

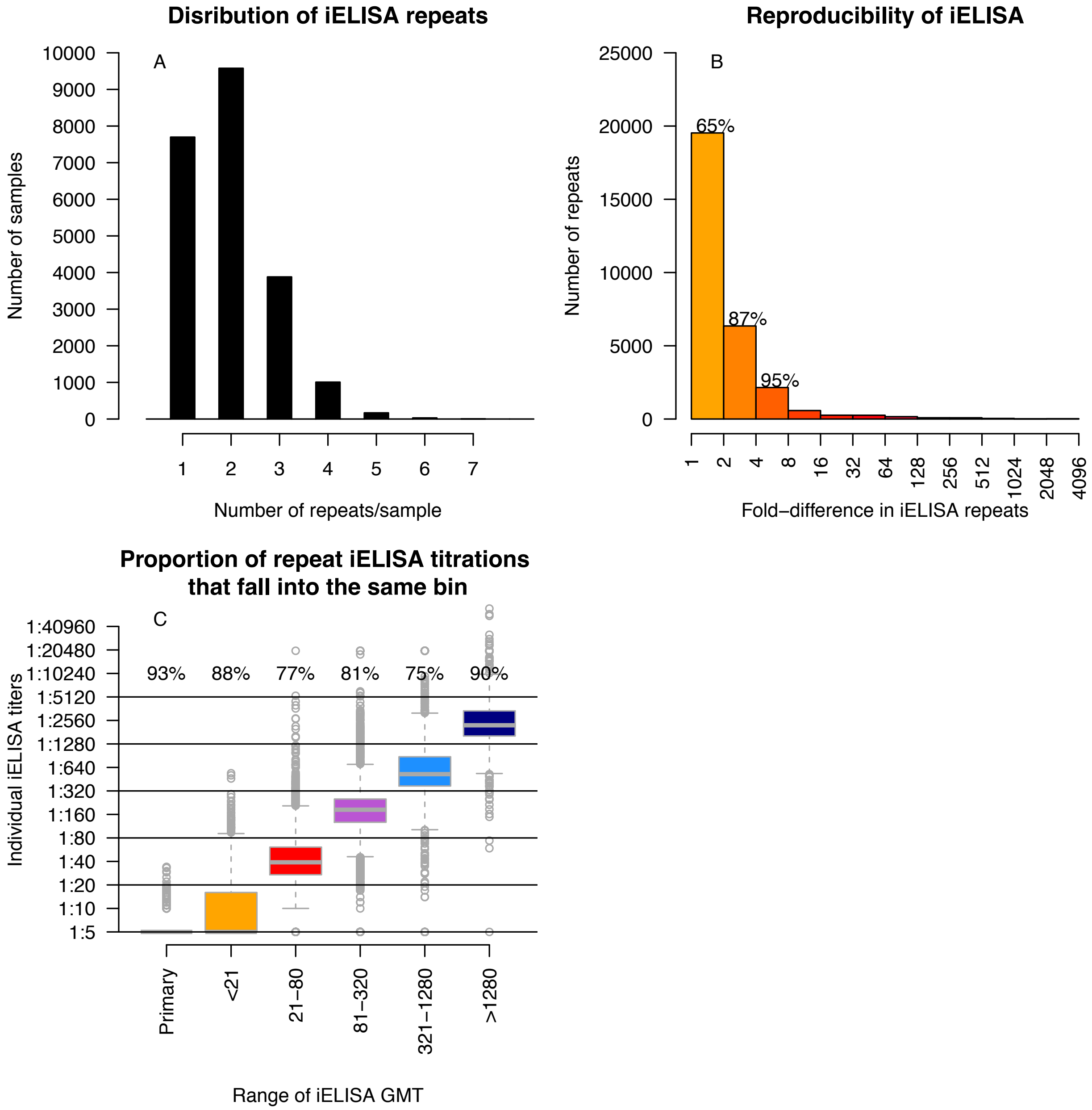

**Fig. S4.**

Comparison of DENV-Ab titers for samples (n=762) in which both the binding (iELISA) and neutralizing (Raji-DCSIGN RVP assay) antibodies were measured. Figures show comparisons of iELISA titers to the neutralizing antibody ($NT_{50}$) titer to each DENV type (DENV1, DENV2, DENV3, or DENV4), or the mean and median $NT_{50}$ titers to DENV1-4. Black line shows bivariate linear-errors-in-variables regression line. Slope (s) and intercept (i) with 95% probability intervals are shown.

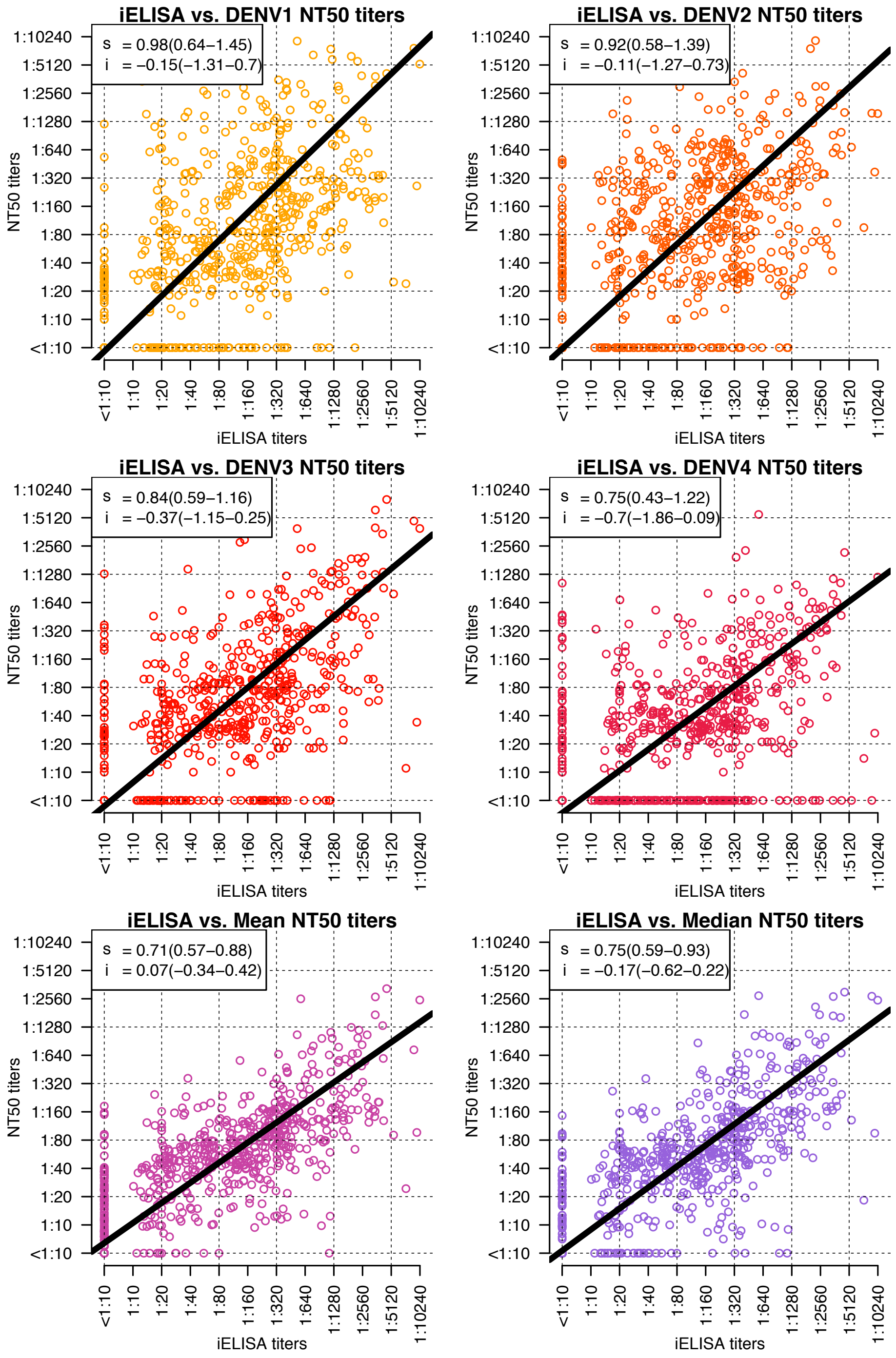

**Fig. S5.**

Comparison of DENV-Ab titers for samples (n=762) in which both binding (iELISA) and neutralizing (Raji-DCSIGN RVP assay) antibodies were measured, stratified by primary infecting DENV type. Neutralizing responses to DENV1-4 for individuals with post-primary DENV1 infections (**A**) and post-primary DENV2 infections (**B**). Dots show titer measurements; lines are linear-error-in-variables regression lines: DENV1, yellow; DENV2, red; DENV3, purple; and DENV4, blue.

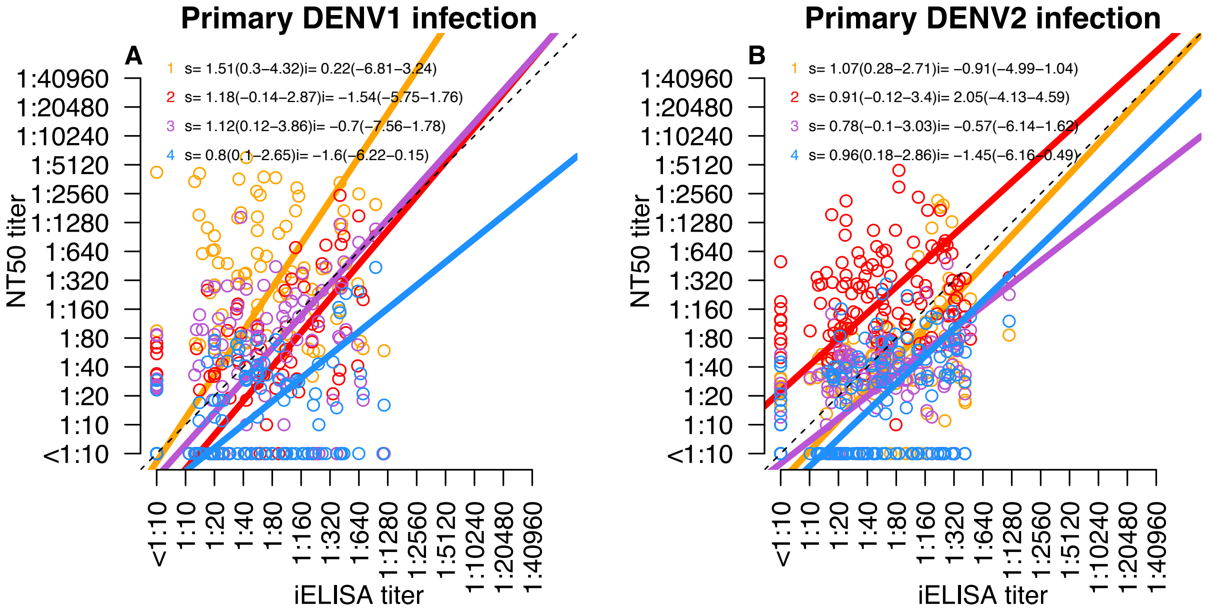

**Fig. S6.**

Longitudinal analyses of the hazard of severe dengue disease or any dengue case by pre-existing DENV-Ab titer in the Pediatric Dengue Cohort Study, controlling for either age or number of previous infections. Hazard ratios with 95% confidence intervals (**A,C,E,G**) and cumulative hazard (**B,D,F,H**) for an average child with severe dengue disease or any dengue case by pre-existing DENV-Ab titer. Cox proportional hazard models were adjusted for sex, epidemic season, and age (**Age only model**) or for sex, epidemic season, and number of previous DENV infections (**Number of previous infections only model**). Average child = female, 2007-2008 epidemic season, age 5-9, and one previous DENV infection.

## Age only model

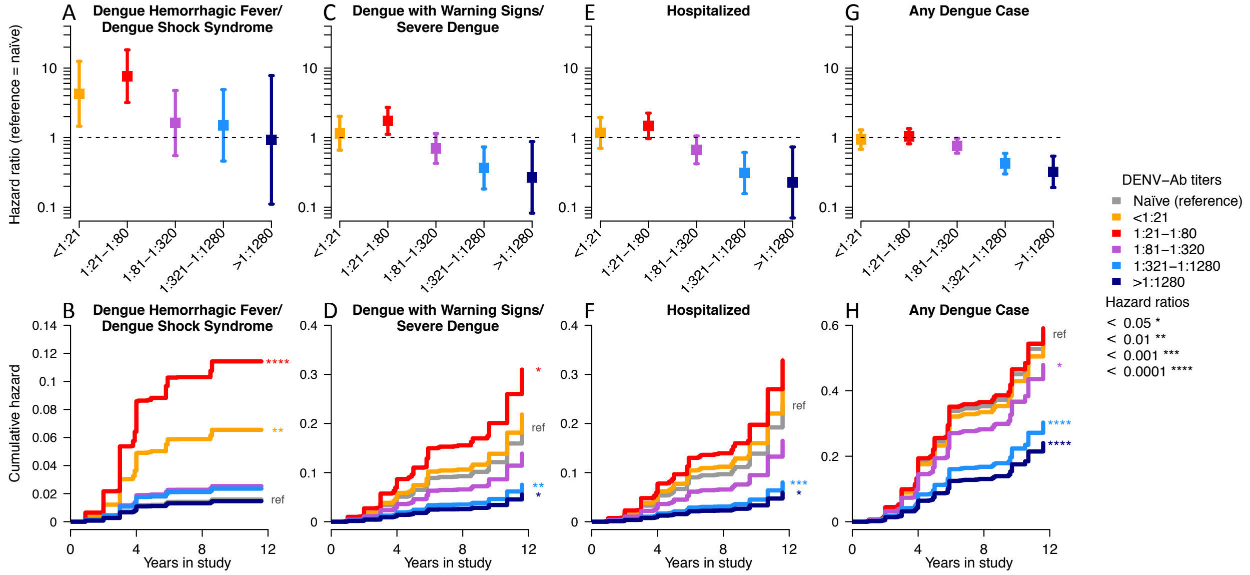


## Number of previous infections only model

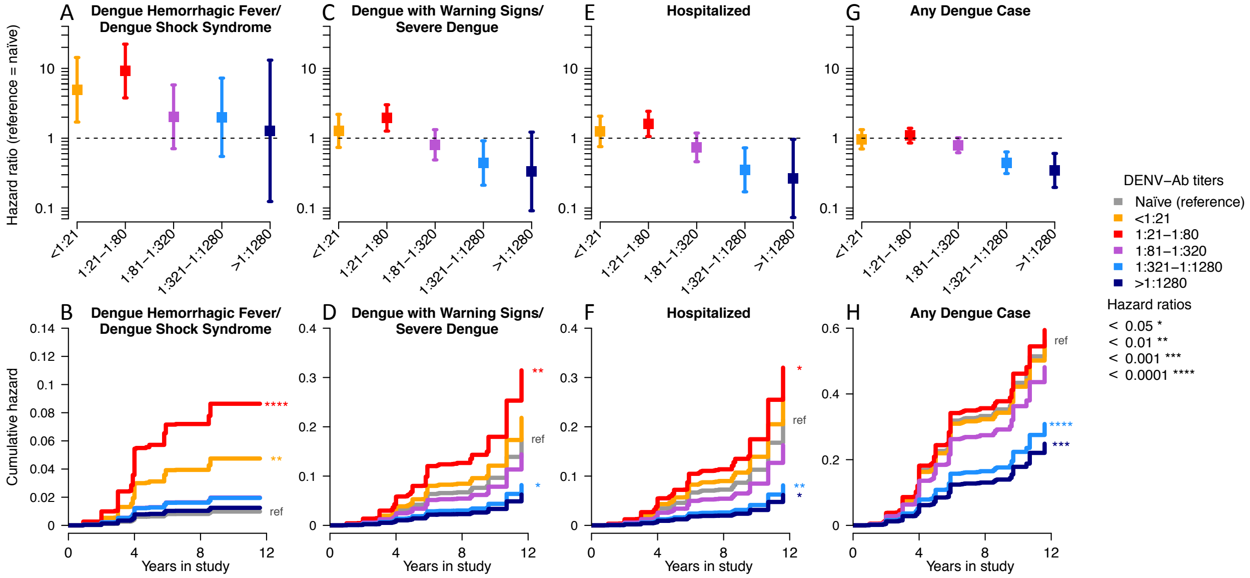

**Fig. S7.**

Longitudinal analyses of the hazard of severe dengue disease or any dengue case by pre-existing DENV-Ab titer in the full Pediatric Dengue Cohort Study, with alternative GMT binning methods. Hazard ratios with 95% confidence intervals (**A,C,E,G**) and cumulative hazard (**B,D,F,H**) for an average child (female, 2007-2008 epidemic season, age 5-9, one previous DENV infection) with severe dengue disease or any dengue case, by pre-existing DENV-Ab titer, binned by **quartiles**, **quintiles**, or **sextiles** of all DENV-Ab titers for non-naïve children in the Pediatric Dengue Cohort Study. Cox proportional hazard models were adjusted for sex, epidemic season, age, and number of previous DENV infections. For the quartiles analysis, the ‘enhancing’ window (1:21-1:80) is split between the first and second quartile.

## DENV-Ab titers binned by quartiles

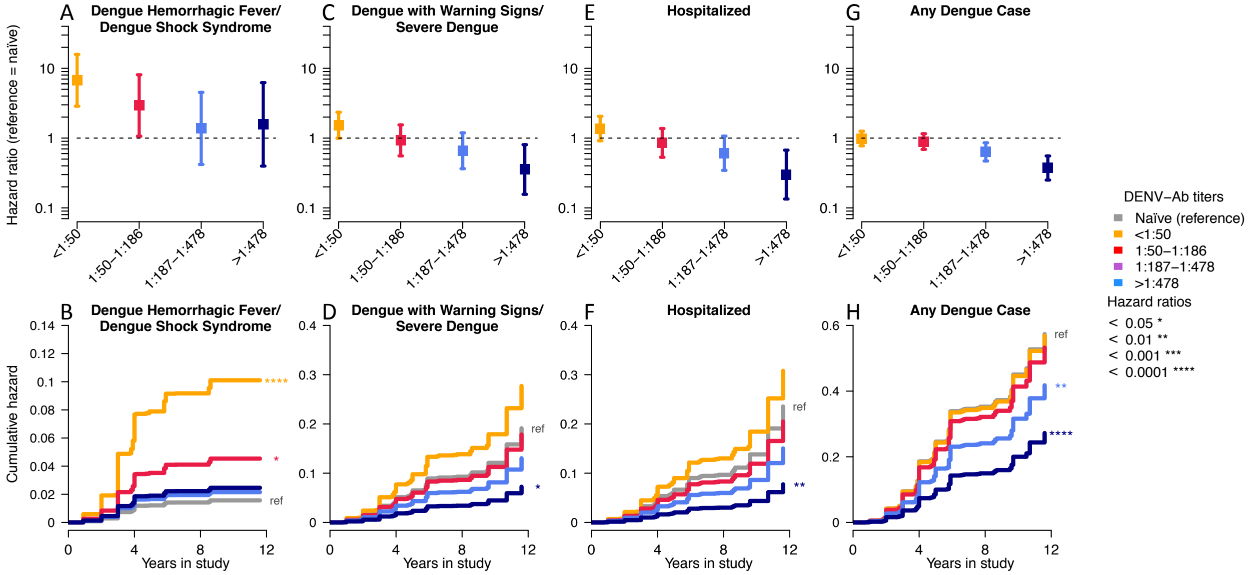


## DENV-Ab titers binned by quintiles

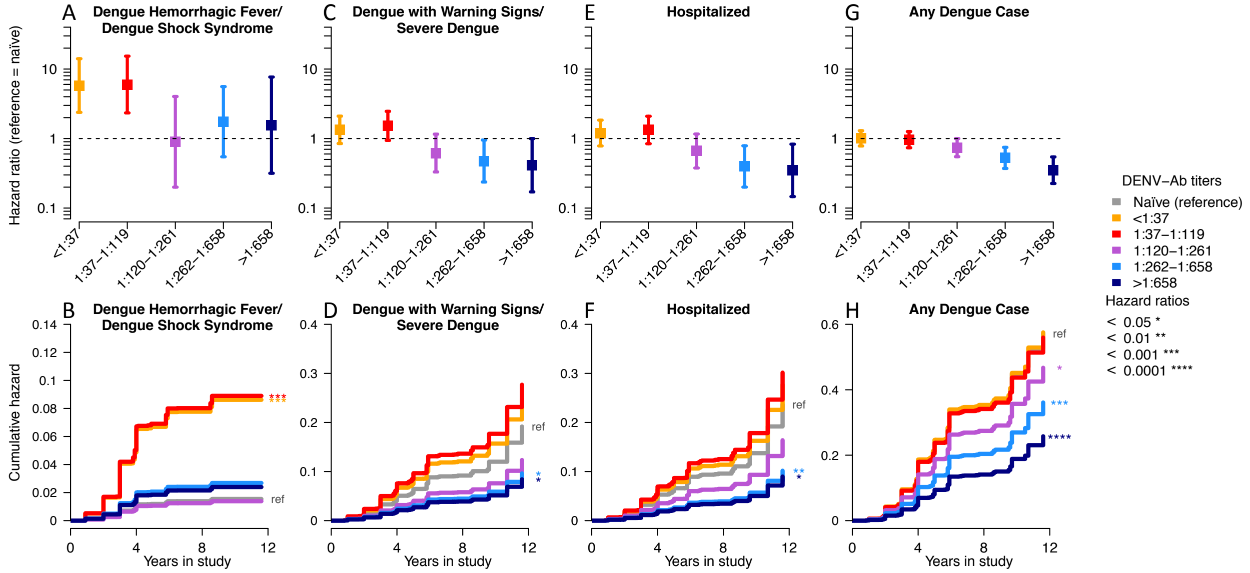

## Fig. S7 continued.

## DENV-Ab titers binned by sextiles

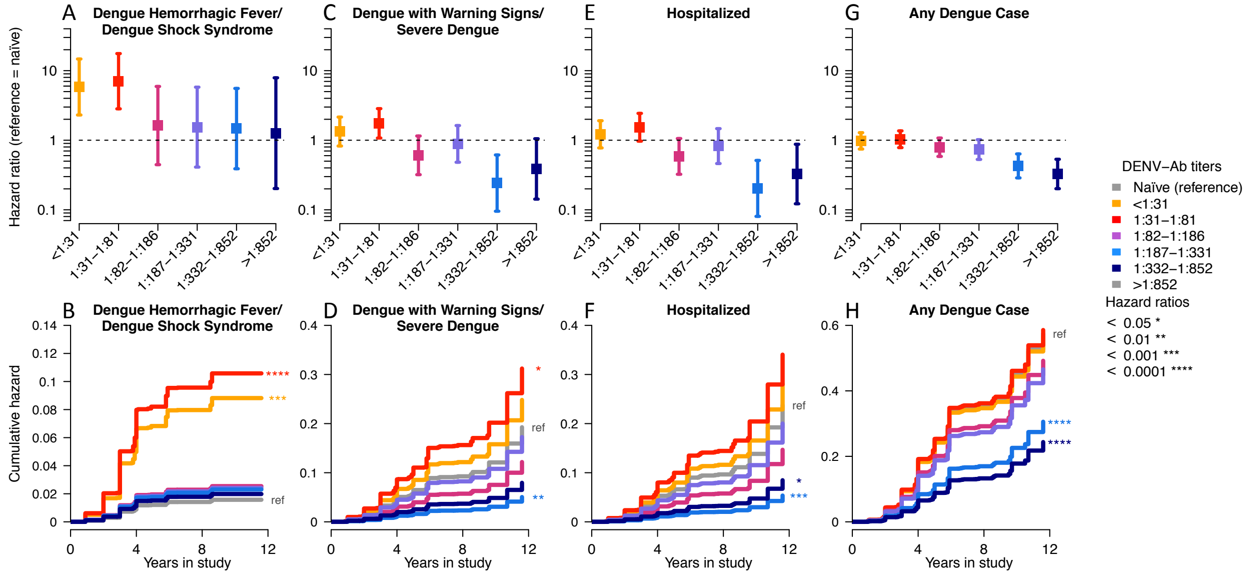

**Fig. S8.**

Longitudinal analyses of the hazard of severe dengue disease or any dengue case by pre-existing DENV-Ab titer in the full Pediatric Dengue Cohort Study, with random sampling of repeat titrations. Shown are hazard ratios and 95% confidence intervals of severe and symptomatic dengue by pre-infection DENV-Ab titer for 50 iELISA titer data sets created by randomly sampling from repeat titrations. Primary infection status was re-classified based on the randomly sampled iELISA titers for each data set.

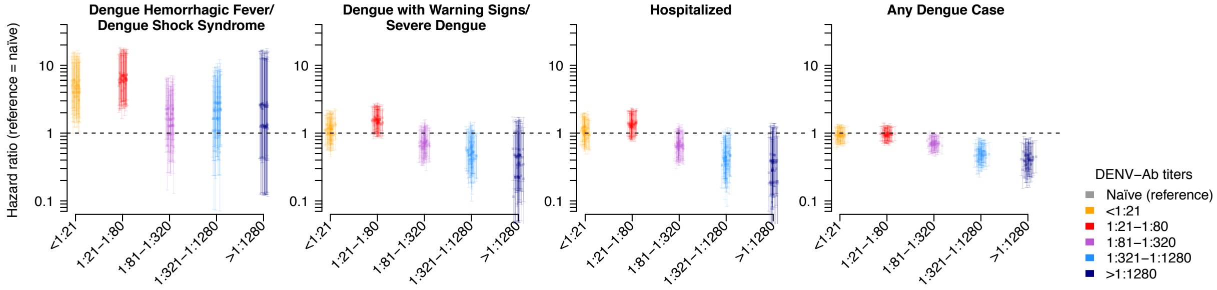

**Fig. S9.**

Analyses of the relationship between DENV-Ab titer and disease outcome with DENV3 infection, excluding individuals with possible prior exposure to DENV3. (**A**) Logistic regression was used to estimate the odds of severe or symptomatic dengue upon DENV3 infection between 2008-2011, by pre-existing DENV-Ab titer. We excluded children born before 1998 (last documented DENV3 circulation), with evidence of two or more infections, or with samples taken after the child experienced a DENV infection between 2008-2011.

A

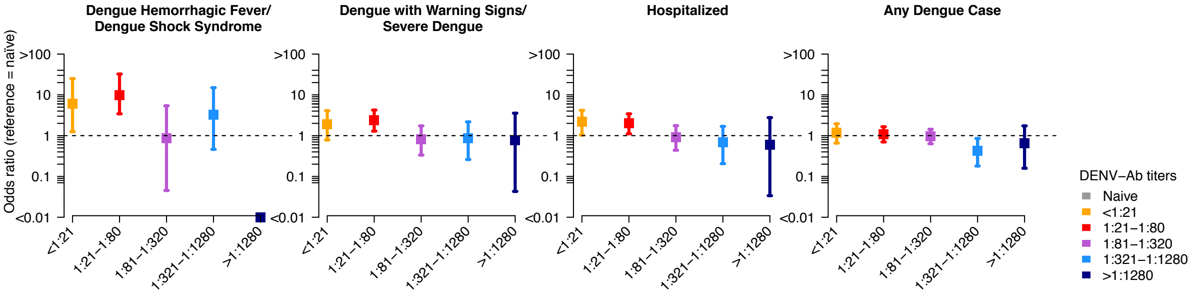

**Fig. S10.**

Spline-based hazard ratio curves for severe dengue disease or any dengue case by pre-existing DENV-Ab titer, including only non-naïve children in the Pediatric Dengue Cohort Study, i.e., children with at least one previous DENV infection. Spline-based hazard models were adjusted for sex, epidemic season, age, and number of previous DENV infections.

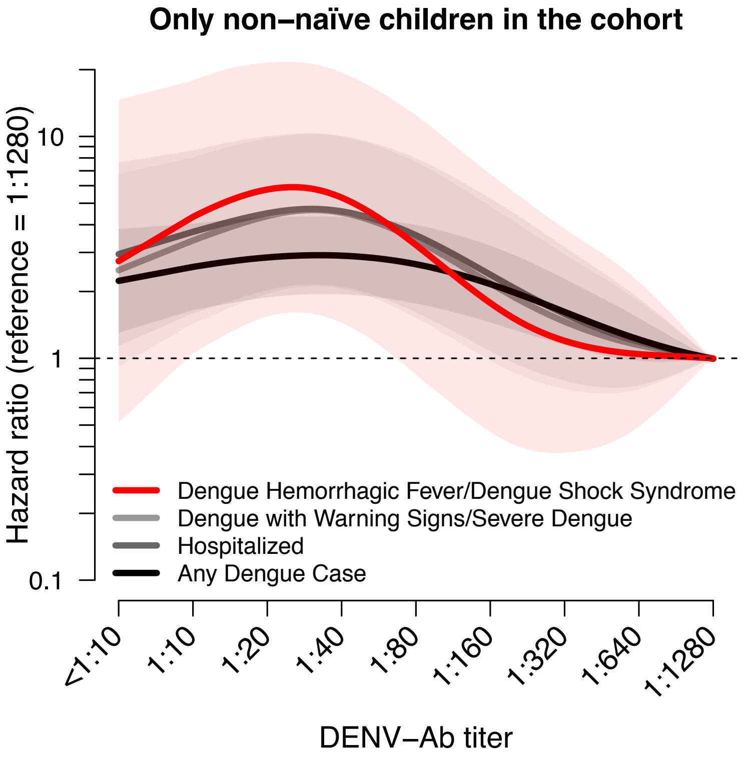

**Fig. S11.**

Comparison of pre-existing DENV-Ab titers in severe or non-severe secondary dengue cases to matched controls drawn randomly from the Pediatric Dengue Cohort Study. Distributions of DENV-Ab titers for severe and non-severe secondary dengue cases and matched controls. The five controls for each case were of the same sex and age, had evidence of prior DENV infection, provided a blood sample within 1-2 months of the case's pre-infection sample, but did not have a dengue case that year. Error bars show one standard deviation, and triangles show distribution medians.

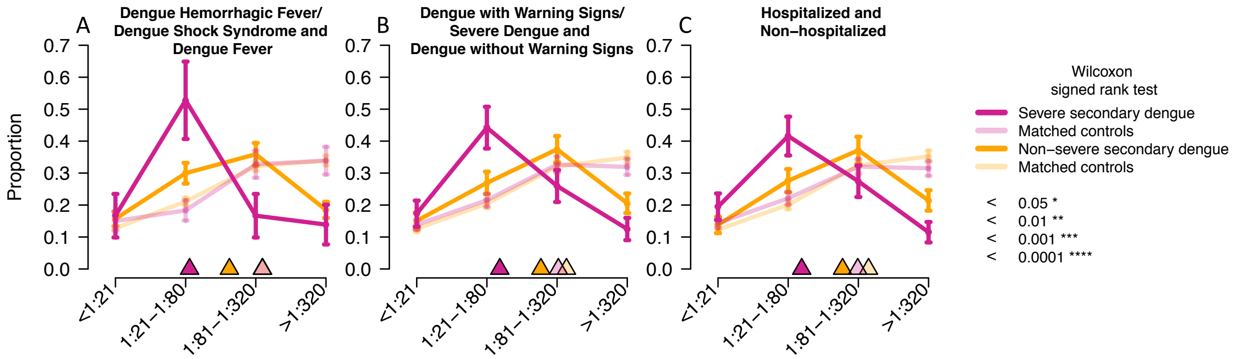

**Fig. S12.**

Comparison of pre-existing DENV-Ab titers for severe or non-severe secondary dengue cases to matched controls drawn randomly from the Pediatric Dengue Cohort Study. (**A-C**) The five controls for each case were of the same sex and age, had evidence of prior DENV infection, provided a blood sample within 1-2 months of the case's pre-infection sample, but did not have a dengue case during their time in the cohort. Conditional logistic regression was used to compare pre-existing DENV-Ab titers of severe cases to those of matched controls and non-severe cases to matched controls, with titers >1:320 as reference. (**D-F**) Distributions of pre-existing DENV-Ab titers in severe and non-severe secondary dengue cases and matched controls (1 control per case). Error bars show one standard deviation, triangles show distribution medians, and brackets indicate significant differences in medians (severe and non-severe cases compared using the Wilcoxon rank sum test, black bracket).

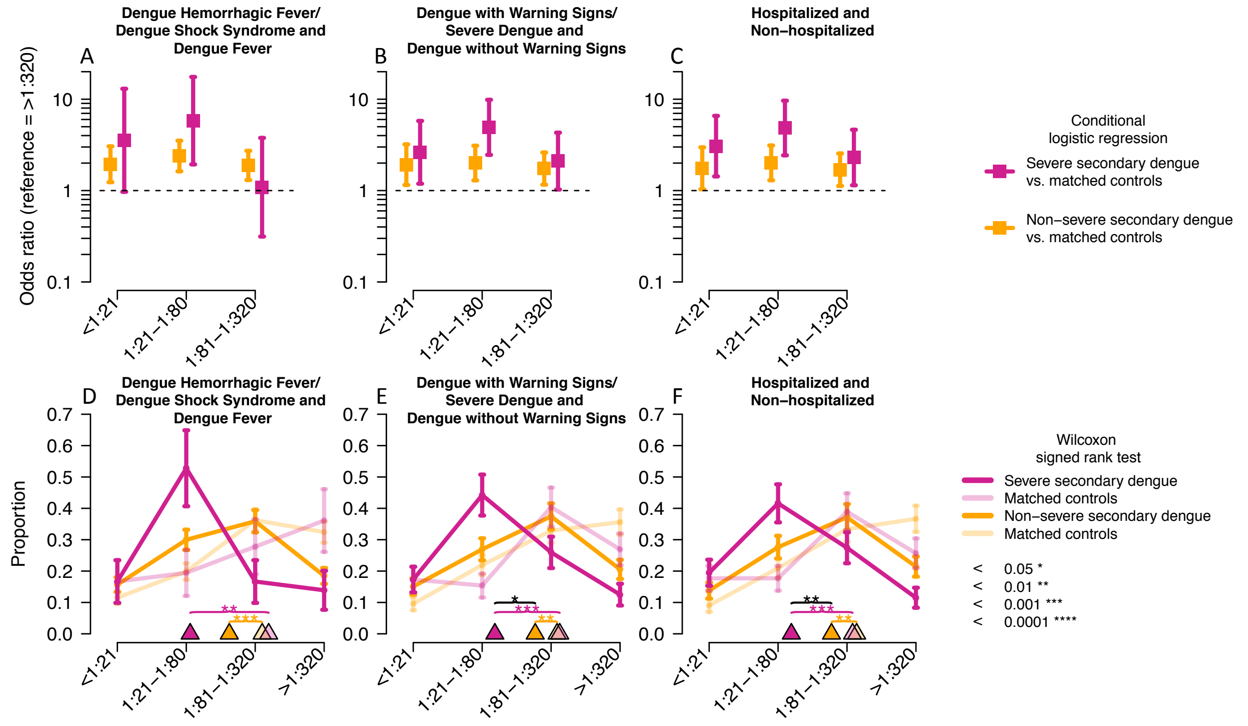

**Tables**

**Table S1.**

Baseline characteristics of the longitudinal cohort.

| | Active cohort | | iELISA titer available* | | Dengue cases | |
|---|---|---|---|---|---|---|
| | N | (%) | N | (%) | N | (%) |
| Total | 8002 | (100%) | 6684 | (100%) | 645 | (100%) |
| Sex | | | | | | |
| Female | 3978 | (49.7%) | 3332 | (49.9%) | 334 | (51.8%) |
| Male | 4024 | (50.3%) | 3352 | (50.1%) | 311 | (48.2%) |
| Year of birth | | | | | | |
| 1993-1998 | 1937 | (24.2%) | 1790 | (26.8%) | 188 | (29.1%) |
| 1999-2004 | 3012 | (37.7%) | 2737 | (40.9%) | 359 | (55.7%) |
| 2005-2010 | 2203 | (27.5%) | 1758 | (26.3%) | 96 | (14.9%) |
| 2011-2015 | 850 | (10.6%) | 399 | (6.0%) | 2 | (0.3%) |
| Year in cohort | | | | | | |
| 2004-2005 | 3712 | | 3396 | | 21 | |
| 2005-2006 | 3681 | | 3643 | | 62 | |
| 2006-2007 | 3556 | | 3681 | | 13 | |
| 2007-2008 | 3675 | | 3574 | | 63 | |
| 2008-2009 | 3934 | | 3836 | | 22 | |
| 2009-2010 | 3837 | | 3778 | | 174 | |
| 2010-2011 | 3685 | | 3629 | | 95 | |
| 2011-2012 | 3363 | | 2826 | | 29 | |
| 2012-2013 | 3741 | | 3527 | | 88 | |
| 2013-2014 | 3676 | | 3181 | | 33 | |
| 2014-2015 | 3912 | | 3237 | | 13 | |
| 2015-2016 | 3733 | | 2994 | | 32 | |

**Table S2.**
Screening for monoclonal antibodies that compete with the conjugated antibody in the iELISA.

| Monoclonal antibody (Ref.) | DENV type specificity | Protein, domain, epitope | iELISA positive* |
|---|---|---|---|
| **1C19** (*83*) | **D1,2,3** | **E, DII, BC loop** | ++ |
| **1M7** (*83*) | **D1,2,3** | **E, DII, fusion loop** | ++ |
| 1F4 (*79*) | D1 | E, DI | - |
| 1F4 Fab (*79*) | D1 | E, DI | - |
| 2B7 (*7*) | D1,2,3,4 | NS1 (inhibits TEER) | - |
| 2D22 (*80*) | D2 | E, DI/DII/DIII | - |
| 2D22 Fab (*80*) | D2 | E, DI/DII/DIII | - |
| **2H2** (*78*) | **D1,2,3,4** | **prM** | ++ |
| **4G2** (*77*) | **D1,2,3,4** | **E, DII, fusion loop** | ++ |
| 5J7 (*81*) | D3 | E, DI/II, hinge | - |
| 5J7 Fab (*81*) | D3 | E, DI/II, hinge | - |
| E111 (*103*) | D1 | E, DIII, CC loop | - |
| **DV2-87** (*84*) | **D2** | **E, DIII, CC loop** | ++ |
| **E1D8** (*85*) | **D1,2,3,4** | **NS3** | ++ |
| EDE A11 (*82*) | D1,2,3,4 | E, EDE2 (N67&N153) | - |
| EDE B7 (*82*) | D1,2,3,4 | E, EDE2 (N67&N153) | - |
| EDE C10 (*82*) | D1,2,3,4 | E, EDE1 (N67) | - |
| EDE C8 (*82*) | D1,2,3,4 | E, EDE1 (N67) | - |

*Antibody inhibition at 0.1 (+) or 0.01 (++) mg/mL.

**Table S3.**

Comparison of DENV-Ab titers in samples in which both the binding (iELISA) antibodies and neutralizing antibodies were measured. (**A**) Raji-DCSIGN RVP based neutralization assay (n=762 samples). (**B**) Single-dilution percent neutralization in the Plaque Reduction Neutralization Test on BHK cells (n=2082 samples).

| **A.** | Pearson's correlation coefficient (95%CI) |
|---|---|
| iELISA vs. DENV1 $NT_{50}$ titer | 0.659 (0.617 - 0.698) |
| iELISA vs. DENV2 $NT_{50}$ titer | 0.644 (0.600 - 0.684) |
| iELISA vs. DENV3 $NT_{50}$ titer | 0.709 (0.672 - 0.743) |
| iELISA vs. DENV4 $NT_{50}$ titer | 0.586 (0.537 - 0.631) |
| iELISA vs. Mean $NT_{50}$ titers | 0.803 (0.776 - 0.827) |
| iELISA vs. Median $NT_{50}$ titers | 0.796 (0.768 - 0.82) |

| **B.** | Pearson's correlation coefficient (95%CI) |
|---|---|
| iELISA vs. Mean $NT_{50}$ titers | 0.727 (0.706 - 0.747) |
| iELISA vs. Median $NT_{50}$ titers | 0.679 (0.655 - 0.701) |

**Table S4.**

Characteristics of dengue cases included in the present study.

| | Primary dengue cases | | Secondary dengue cases | |
|---|---|---|---|---|
| | N | % | N | % |
| Total | 295 | (100%) | 323 | (100%) |
| Age at sampling (mean, sd) | 7.7, 3 | | 9.6, 2.9 | |
| Female (%) | 169 | (57.3%) | 156 | (48.3%) |
| Male (%) | 126 | (42.7%) | 167 | (51.7%) |
| Number of previous infections (%) | | | | |
| 0 | 295 | (100%) | 0 | (0%) |
| 1 | 0 | (0%) | 260 | (80.5%) |
| ≥2 | 0 | (0%) | 63 | (19.5%) |
| Disease severity | | | | |
| DHF/DSS (%) | 8 | (2.7%) | 36 | (11.1%) |
| Dengue with Warning Signs/Severe Dengue (%) | 70 | (23.7%) | 104 | (32.2%) |
| Hospitalized (%) | 86 | (29.2%) | 113 | (35.0%) |
| RT-PCR-confirmed DENV (%) | | | | |
| DENV1 | 83 | (28.1%) | 67 | (20.7%) |
| DENV2 | 49 | (16.6%) | 109 | (33.7%) |
| DENV3 | 127 | (43.1%) | 120 | (37.2%) |
| No DENV type identified | 36 | (12.2%) | 27 | (8.4%) |

**Table S5.**

Comparison of dengue case classification using 1997 WHO criteria, 2009 WHO criteria, and a clinical management score.

**Primary**

**Clinical management score** = I (non-hospitalized)

| | **2009 WHO criteria** | |
|---|---|---|
| **1997 WHO criteria** | Dengue without Warnings Signs | Dengue with Warning Signs/Severe Dengue |
| DF | 209 | 0 |
| DHF/DSS | 0 | 0 |

**Clinical management score** = II/III (hospitalized/intensive care)

| | **2009 WHO criteria** | |
|---|---|---|
| **1997 WHO criteria** | Dengue without Warnings Signs | Dengue with Warning Signs/Severe Dengue |
| DF | 16 | 62 |
| DHF/DSS | 0 | 8 |

**Secondary**

**Clinical management score** = I (non-hospitalized)

| | **2009 WHO criteria** | |
|---|---|---|
| **1997 WHO criteria** | Dengue without Warnings Signs | Dengue with Warning Signs/Severe Dengue |
| DF | 209 | 0 |
| DHF/DSS | 0 | 1 |

**Clinical management score** = II/III (hospitalized/intensive care)

| | **2009 WHO criteria** | |
|---|---|---|
| **1997 WHO criteria** | Dengue without Warnings Signs | Dengue with Warning Signs/Severe Dengue |
| DF | 9 | 69 |
| DHF/DSS | 1 | 34 |

**Table S6.**

Hazard ratio estimates for Dengue Hemorrhagic Fever/Dengue Shock Syndrome in the Pediatric Dengue Cohort.

| Covariate | HR | [95%CI] |
|---|---|---|
| Naïve as reference | | |
| <1:21 | 4.261 | [ 1.459 - 12.445 ] |
| 1:21-1:80 | 7.641 | [ 3.194 - 18.281 ] |
| 1:81-1:320 | 1.622 | [ 0.578 - 4.552 ] |
| 1:321-1:1280 | 1.511 | [ 0.440 - 5.190 ] |
| >1:1280 | 0.939 | [ 0.090 - 9.827 ] |
| 2007-2008 as reference | | |
| 2004-2005 | 0.000 | [ 0.000 - 0.000 ] |
| 2005-2006 | 0.000 | [ 0.000 - 0.000 ] |
| 2006-2007 | 3.474 | [ 0.245 - 49.304 ] |
| 2008-2009 | 0.686 | [ 0.194 - 2.433 ] |
| 2009-2010 | 1.083 | [ 0.295 - 3.978 ] |
| 2010-2011 | 24.701 | [ 4.293 - 142.108 ] |
| 2011-2012 | 0.000 | [ 0.000 - 0.000 ] |
| 2012-2013 | 0.483 | [ 0.121 - 1.926 ] |
| 2013-2014 | 0.309 | [ 0.030 - 3.146 ] |
| 2014-2015 | 0.000 | [ 0.000 - 0.000 ] |
| 2015-2016 | 0.000 | [ 0.000 - 0.000 ] |
| Ages 2-4 as reference | | |
| 5-9 | 3.932 | [ 0.490 - 31.551 ] |
| 10-14 | 6.249 | [ 0.742 - 52.590 ] |
| 1 infection as reference | | |
| ≥2 infections | 0.983 | [ 0.353 - 2.738 ] |
| Females as reference | | |
| Male | 0.978 | [ 0.547 - 1.749 ] |

**Table. S7.**

Antibody decay in the Pediatric Dengue Cohort Study. (**A**) Results of a mixed-effects linear model to estimate DENV-Ab decay rates, controlling for number of previous infections and allowing for differences in individual intercepts and slopes with random variables. (**B**) Predicted proportion of children with iELISA titers within the enhancing window (1:21-1:80) or the protective DENV-Ab titer window (>1:80) by number of years after their previous infection.

| **Fixed effects** | **Est (95%CI)** |
|---|---|
| Intercept* | 7.8 (7.72-7.88) |
| Previous infections (ref=1)* | |
| 2 | 1.69 (1.55-1.84) |
| >2 | 2.69 (2.36-3.03) |
| *DENV-Ab decay (slope)* † | *-0.25 (-0.26--0.24)* |
| **Random effects** | **SD** |
| Intercept* | 2.27 |
| DENV-Ab decay† | 0.29 |

*Unit = $\log_2$(titer).
†Unit = $\log_2$(titer)/year.

| Years post-infection | Enhancing window (%1:21-1:80) | Protection window (% >1:80) |
|---|---|---|
| 1 | 18 | 77 |
| 2 | 20 | 75 |
| 3 | 22 | 71 |
| 4 | 23 | 68 |
| 5 | 25 | 64 |
| 6 | 28 | 59 |
| 7 | 30 | 55 |
| 8 | 30 | 51 |

**Table S8.**

Estimates of iELISA titer decay rates by subsequent dengue disease outcome using a mixed-effects linear model to control for number of previous infections, account for differences in individual intercepts and slopes, and measure interaction between subsequent severe dengue infection and antibody decay rates.

| | Subsequent disease severity: Est (95%CI) | | | |
|---|---|---|---|---|
| **Fixed effects** | DHF/DSS | Dengue+Warning Signs/Severe Dengue | Hospitalized Dengue | Any Dengue Case |
| Intercept* | 7.81 (7.73-7.89) | 7.82 (7.74-7.9) | 7.82 (7.74-7.9) | 7.85 (7.77-7.93) |
| Previous infections (ref=1)* | | | | |
| 2 | 1.69 (1.55-1.83) | 1.69 (1.54-1.83) | 1.69 (1.54-1.83) | 1.68 (1.54-1.82) |
| >2 | 2.68 (2.35-3.02) | 2.67 (2.34-3.01) | 2.67 (2.34-3) | 2.66 (2.33-2.99) |
| *Subsequent severe infection*,†* | *-0.89 (-1.71--0.08)* | *-0.75 (-1.23--0.27)* | *-0.83 (-1.29--0.36)* | *-0.64 (-0.92--0.36)* |
| DENV-Ab decay (Slope) ‡ | -0.25 (-0.26--0.24) | -0.25 (-0.26--0.24) | -0.25 (-0.26--0.24) | -0.25 (-0.26--0.24) |
| *DENV-Ab decay by subsequent severe infection†,‡* | *-0.01 (-0.15-0.14)* | *-0.01 (-0.1-0.07)* | *0.00 (-0.08-0.08)* | *0.01 (-0.04-0.06)* |
| **Random effects** | **SD** | **SD** | **SD** | **SD** |
| Intercept* | 2.27 | 2.27 | 2.27 | 2.27 |
| DENV-Ab decay‡ | 0.29 | 0.29 | 0.29 | 0.29 |

*Unit = $\log_2$(titer).
†The reference group consisted of children without subsequent severe dengue cases of that disease classification (DHF/SSS, etc.).
‡Unit = $\log_2$(titer)/year.

**Table S9.**

Hazard ratio estimates for Dengue + Warning Signs/Severe Dengue in the Pediatric Dengue Cohort Study.

| Covariate | HR | [95%CI] |
|---|---|---|
| Naïve as reference | | |
| <1:21 | 1.158 | [ 0.663 - 2.023 ] |
| 1:21-1:80 | 1.747 | [ 1.114 - 2.739 ] |
| 1:81-1:320 | 0.707 | [ 0.429 - 1.167 ] |
| 1:321-1:1280 | 0.375 | [ 0.180 - 0.782 ] |
| >1:1280 | 0.278 | [ 0.076 - 1.021 ] |
| 2007-2008 as reference | | |
| 2004-2005 | 1.170 | [ 0.033 -41.411 ] |
| 2005-2006 | 0.511 | [ 0.037 - 7.158 ] |
| 2006-2007 | 0.715 | [ 0.117 - 4.352 ] |
| 2008-2009 | 0.227 | [ 0.071 - 0.731 ] |
| 2009-2010 | 1.481 | [ 0.679 - 3.228 ] |
| 2010-2011 | 9.594 | [ 3.721 -24.740 ] |
| 2011-2012 | 2.246 | [ 0.621 - 8.125 ] |
| 2012-2013 | 1.434 | [ 0.551 - 3.735 ] |
| 2013-2014 | 0.914 | [ 0.344 - 2.427 ] |
| 2014-2015 | 0.000 | [ 0.000 - 0.000 ] |
| 2015-2016 | 0.090 | [ 0.018 - 0.448 ] |
| Ages 2-4 as reference | | |
| 5-9 | 2.782 | [ 1.248 - 6.200 ] |
| 10-14 | 3.831 | [ 1.573 - 9.332 ] |
| 1 infection as reference | | |
| ≥2 infections | 0.947 | [ 0.548 - 1.638 ] |
| Females as reference | | |
| Male | 0.820 | [ 0.611 - 1.102 ] |

**Table S10.**

Hazard ratio estimates for Hospitalized Dengue in the Pediatric Dengue Cohort Study.

| Covariate | HR | [95%CI] |
|---|---|---|
| Naïve as reference | | |
| <1:21 | 1.170 | [ 0.702 - 1.952 ] |
| 1:21-1:80 | 1.481 | [ 0.967 - 2.267 ] |
| 1:81-1:320 | 0.673 | [ 0.420 - 1.077 ] |
| 1:321-1:1280 | 0.314 | [ 0.152 - 0.649 ] |
| >1:1280 | 0.233 | [ 0.064 - 0.845 ] |
| 2007-2008 as reference | | |
| 2004-2005 | 1.324 | [ 0.037 -47.480] |
| 2005-2006 | 0.574 | [ 0.044 - 7.428 ] |
| 2006-2007 | 0.669 | [ 0.123 - 3.640 ] |
| 2008-2009 | 0.163 | [ 0.050 - 0.530 ] |
| 2009-2010 | 1.933 | [ 0.953 - 3.920 ] |
| 2010-2011 | 7.983 | [ 3.225 -19.758] |
| 2011-2012 | 1.649 | [ 0.486 - 5.592 ] |
| 2012-2013 | 1.441 | [ 0.583 - 3.561 ] |
| 2013-2014 | 1.010 | [ 0.428 - 2.384 ] |
| 2014-2015 | 0.000 | [ 0.000 - 0.000 ] |
| 2015-2016 | 0.073 | [ 0.015 - 0.358 ] |
| Ages 2-4 as reference | | |
| 5-9 | 2.411 | [ 1.161 - 5.006 ] |
| 10-14 | 2.948 | [ 1.301 - 6.678 ] |
| 1 infection as reference | | |
| ≥2 infections | 0.963 | [ 0.570 - 1.624 ] |
| Females as reference | | |
| Male | 0.883 | [ 0.670 - 1.163 ] |

**Table S11.**

Hazard ratio estimates for Any Dengue Case in the Pediatric Dengue Cohort Study.

| **Covariate** | **HR** | **[95%CI]** |
|---|---|---|
| Naïve as reference | | |
| <1:21 | 0.937 | [ 0.680 - 1.292 ] |
| 1:21-1:80 | 1.048 | [ 0.816 - 1.344 ] |
| 1:81-1:320 | 0.765 | [ 0.594 - 0.986 ] |
| 1:321-1:1280 | 0.425 | [ 0.295 - 0.612 ] |
| >1:1280 | 0.325 | [ 0.185 - 0.571 ] |
| 2007-2008 as reference | | |
| 2004-2005 | 5.162 | [ 1.438 - 18.527 ] |
| 2005-2006 | 4.244 | [ 2.432 - 7.406 ] |
| 2006-2007 | 0.469 | [ 0.266 - 0.825 ] |
| 2008-2009 | 0.217 | [ 0.122 - 0.386 ] |
| 2009-2010 | 0.914 | [ 0.637 - 1.313 ] |
| 2010-2011 | 11.832 | [ 7.160 - 19.551 ] |
| 2011-2012 | 2.638 | [ 1.324 - 5.253 ] |
| 2012-2013 | 2.364 | [ 1.398 - 3.997 ] |
| 2013-2014 | 0.590 | [ 0.359 - 0.970 ] |
| 2014-2015 | 0.133 | [ 0.065 - 0.269 ] |
| 2015-2016 | 0.142 | [ 0.080 - 0.252 ] |
| Ages 2-4 as reference | | |
| 5-9 | 1.244 | [ 0.901 - 1.716 ] |
| 10-14 | 1.355 | [ 0.916 - 2.003 ] |
| 1 infection as reference | | |
| ≥2 infections | 0.984 | [ 0.720 - 1.345 ] |
| Females as reference | | |
| Male | 0.879 | [ 0.751 - 1.029 ] |

**Table S12.**

Odds ratios for Dengue Hemorrhagic Fever/Dengue Shock Syndrome vs. Dengue Fever.

| Covariate | OR | [95%CI] |
|---|---|---|
| Naïve as reference | | |
| <1:21 | 5.04 | [ 1.48 - 16.55 ] |
| 1:21-1:80 | 8.60 | [ 3.41 - 23.59 ] |
| 1:81-1:320 | 2.42 | [ 0.71 - 7.83 ] |
| >1:320 | 4.06 | [ 1.01 - 15.41 ] |
| 2007-2008 as reference | | |
| 2004-2005 | 0.00 | [ 0.00 - Inf ] |
| 2005-2006 | 0.00 | [ 0.00 - Inf ] |
| 2006-2007 | 2.15 | [ 0.26 - 12.84 ] |
| 2008-2009 | 4.62 | [ 0.75 - 25.85 ] |
| 2009-2010 | 1.38 | [ 0.34 - 5.41 ] |
| 2010-2011 | 1.38 | [ 0.34 - 5.41 ] |
| 2011-2012 | 0.00 | [ 0.00 - Inf ] |
| 2012-2013 | 0.77 | [ 0.09 - 6.51 ] |
| 2013-2014 | 0.43 | [ 0.02 - 3.59 ] |
| 2014-2015 | 0.00 | [ NA - Inf ] |
| 2015-2016 | 0.00 | [ 0.00 - Inf ] |
| Females as reference | | |
| Male | 1.01 | [ 0.52 - 1.97 ] |
| 1 infection as reference | | |
| ≥2 infections | 0.71 | [ 0.24 - 1.82 ] |
| Ages 2-4 as reference | | |
| 5-9 | 1.59 | [ 0.46 - 7.45 ] |
| 10-14 | 1.85 | [ 0.51 - 9.04 ] |
| DENV3 as reference | | |
| DENV1 | 0.84 | [ 0.15 - 3.57 ] |
| DENV2 | 1.21 | [ 0.37 - 3.52 ] |
| DENV type unknown | 0.00 | [ 0.00 - Inf ] |

**Table S13.**

Odds ratios for Dengue with Warning Signs/Severe Dengue vs. Dengue without Warning Signs.

| Covariate | OR | [95%CI] |
|---|---|---|
| Naïve as reference | | |
| <1:21 | 1.439 | [ 0.704 - 2.893 ] |
| 1:21-1:80 | 3.051 | [ 1.729 - 5.438 ] |
| 1:81-1:320 | 1.094 | [ 0.598 - 1.971 ] |
| >1:320 | 1.081 | [ 0.461 - 2.449 ] |
| 2007-2008 as reference | | |
| 2004-2005 | 0.818 | [ 0.109 - 3.987 ] |
| 2005-2006 | 0.075 | [ 0.004 - 0.422 ] |
| 2006-2007 | 0.851 | [ 0.116 - 4.026 ] |
| 2008-2009 | 0.804 | [ 0.187 - 3.127 ] |
| 2009-2010 | 1.853 | [ 0.697 - 4.986 ] |
| 2010-2011 | 1.190 | [ 0.436 - 3.245 ] |
| 2011-2012 | 1.173 | [ 0.303 - 4.337 ] |
| 2012-2013 | 1.689 | [ 0.532 - 5.446 ] |
| 2013-2014 | 3.606 | [ 1.144 - 11.608 ] |
| 2014-2015 | 0.000 | [ 0 - Inf ] |
| 2015-2016 | 0.894 | [ 0.247 - 2.879 ] |
| Females as reference | | |
| Male | 0.927 | [ 0.628 - 1.366 ] |
| 1 infection as reference | | |
| ≥2 infections | 0.662 | [ 0.336 - 1.278 ] |
| Ages 2-4 as reference | | |
| 5-9 | 1.863 | [ 0.955 - 3.850 ] |
| 10-14 | 2.945 | [ 1.426 - 6.404 ] |
| DENV3 as reference | | |
| DENV1 | 0.491 | [ 0.218 - 1.062 ] |
| DENV2 | 0.558 | [ 0.251 - 1.207 ] |
| DENV type unknown | 0.143 | [ 0.032 - 0.444 ] |

**Table S14.**
Odds ratios for Hospitalized vs. Non-hospitalized Dengue Cases.

| Covariate | OR | [95%CI] |
|---|---|---|
| Naïve as reference | | |
| <1:21 | 1.555 | [ 0.769 - 3.143 ] |
| 1:21-1:80 | 2.443 | [ 1.379 - 4.372 ] |
| 1:81-1:320 | 1.002 | [ 0.554 - 1.792 ] |
| >1:320 | 0.814 | [ 0.344 - 1.858 ] |
| 2007-2008 as reference | | |
| 2004-2005 | 0.694 | [ 0.092 - 3.384 ] |
| 2005-2006 | 0.066 | [ 0.003 - 0.376 ] |
| 2006-2007 | 0.762 | [ 0.104 - 3.585 ] |
| 2008-2009 | 0.479 | [ 0.102 - 1.932 ] |
| 2009-2010 | 2.229 | [ 0.851 - 5.930 ] |
| 2010-2011 | 1.298 | [ 0.483 - 3.500 ] |
| 2011-2012 | 1.181 | [ 0.317 - 4.270 ] |
| 2012-2013 | 1.386 | [ 0.442 - 4.399 ] |
| 2013-2014 | 4.931 | [ 1.577 - 16.074 ] |
| 2014-2015 | 0.000 | [ 0 - Inf ] |
| 2015-2016 | 0.900 | [ 0.250 - 2.877 ] |
| Females as reference | | |
| Male | 1.063 | [ 0.725 - 1.559 ] |
| 1 infection as reference | | |
| ≥2 infections | 0.665 | [ 0.340 - 1.283 ] |
| Ages 2-4 as reference | | |
| 5-9 | 2.028 | [ 1.068 - 4.016 ] |
| 10-14 | 2.921 | [ 1.444 - 6.137 ] |
| DENV3 as reference | | |
| DENV1 | 0.514 | [ 0.237 - 1.088 ] |
| DENV2 | 0.480 | [ 0.218 - 1.032 ] |
| DENV type unknown | 0.110 | [ 0.025 - 0.339 ] |

**References and Notes**


1. S. Bhatt, P. W. Gething, O. J. Brady, J. P. Messina, A. W. Farlow, C. L. Moyes, J. M. Drake, J. S. Brownstein, A. G. Hoen, O. Sankoh, M. F. Myers, D. B. George, T. Jaenisch, G. R. Wint, C. P. Simmons, T. W. Scott, J. J. Farrar, S. I. Hay, The global distribution and burden of dengue. *Nature* **496**, 504–507 (2013). doi:10.1038/nature12060 Medline

2. World Health Organization (WHO)/Special Programme for Research and Training in Tropical Diseases (TDR), "Dengue: Guidelines for diagnosis, treatment, prevention and control" (WHO, 2009).

3. WHO, "Dengue haemorrhagic fever: Diagnosis, treatment, prevention and control" (WHO, ed. 2, 1997).

4. N. Sangkawibha, S. Rojanasuphot, S. Ahandrik, S. Viriyapongse, S. Jatanasen, V. Salitul, B. Phanthumachinda, S. B. Halstead, Risk factors in dengue shock syndrome: A prospective epidemiologic study in Rayong, Thailand. I. The 1980 outbreak. *Am. J. Epidemiol.* **120**, 653–669 (1984). doi:10.1093/oxfordjournals.aje.a113932 Medline

5. M. G. Guzman, E. Harris, Dengue. *Lancet* **385**, 453–465 (2015). doi:10.1016/S0140-6736(14)60572-9 Medline

6. T. T. Wang, J. Sewatanon, M. J. Memoli, J. Wrammert, S. Bournazos, S. K. Bhaumik, B. A. Pinsky, K. Chokephaibulkit, N. Onlamoon, K. Pattanapanyasat, J. K. Taubenberger, R. Ahmed, J. V. Ravetch, IgG antibodies to dengue enhanced for FcγRIIIA binding determine disease severity. *Science* **355**, 395–398 (2017). doi:10.1126/science.aai8128 Medline

7. P. R. Beatty, H. Puerta-Guardo, S. S. Killingbeck, D. R. Glasner, K. Hopkins, E. Harris, Dengue virus NS1 triggers endothelial permeability and vascular leak that is prevented by NS1 vaccination. *Sci. Transl. Med.* **7**, 304ra141 (2015). doi:10.1126/scitranslmed.aaa3787 Medline

8. R. M. Zellweger, T. R. Prestwood, S. Shresta, Enhanced infection of liver sinusoidal endothelial cells in a mouse model of antibody-induced severe dengue disease. *Cell Host Microbe* **7**, 128–139 (2010). doi:10.1016/j.chom.2010.01.004 Medline

9. K. L. Williams, S. Sukupolvi-Petty, M. Beltramello, S. Johnson, F. Sallusto, A. Lanzavecchia, M. S. Diamond, E. Harris, Therapeutic efficacy of antibodies lacking Fcγ receptor binding against lethal dengue virus infection is due to neutralizing potency and blocking of enhancing antibodies. *PLOS Pathog.* **9**, e1003157 (2013). doi:10.1371/journal.ppat.1003157 Medline

10. S. R. Hadinegoro, J. L. Arredondo-García, M. R. Capeding, C. Deseda, T. Chotpitayasunondh, R. Dietze, H. I. Muhammad Ismail, H. Reynales, K. Limkittikul, D. M. Rivera-Medina, H. N. Tran, A. Bouckenooghe, D. Chansinghakul, M. Cortés, K. Fanouillere, R. Forrat, C. Frago, S. Gailhardou, N. Jackson, F. Noriega, E. Plennevaux, T. A. Wartel, B. Zambrano, M. Saville; CYD-TDV Dengue Vaccine Working Group, Efficacy and long-term safety of a dengue vaccine in regions of endemic disease. *N. Engl. J. Med.* **373**, 1195–1206 (2015). doi:10.1056/NEJMoa1506223 Medline

11. N. M. Ferguson, I. Rodríguez-Barraquer, I. Dorigatti, L. Mier-y-Teran-Romero, D. J. Laydon, D. A. T. Cummings, Benefits and risks of the Sanofi-Pasteur dengue vaccine: Modeling optimal deployment. *Science* **353**, 1033–1036 (2016). doi:10.1126/science.aaf9590 Medline

12. C. P. Simmons, T. N. Chau, T. T. Thuy, N. M. Tuan, D. M. Hoang, N. T. Thien, B. Lien, N. T. Quy, N. T. Hieu, T. T. Hien, C. McElnea, P. Young, S. Whitehead, N. T. Hung, J. Farrar, Maternal antibody and viral factors in the pathogenesis of dengue virus in infants. *J. Infect. Dis.* **196**, 416–424 (2007). doi:10.1086/519170 Medline

13. T. N. Chau, N. T. Quyen, T. T. Thuy, N. M. Tuan, D. M. Hoang, N. T. Dung, B. Lien, N. T. Quy, N. T. Hieu, L. T. Hieu, T. T. Hien, N. T. Hung, J. Farrar, C. P. Simmons, Dengue in Vietnamese infants—Results of infection-enhancement assays correlate with age-related disease epidemiology, and cellular immune responses correlate with disease severity. *J. Infect. Dis.* **198**, 516–524 (2008). doi:10.1086/590117 Medline

14. T. N. Chau, N. T. Hieu, K. L. Anders, M. Wolbers, B. Lien, L. T. Hieu, T. T. Hien, N. T. Hung, J. Farrar, S. Whitehead, C. P. Simmons, Dengue virus infections and maternal antibody decay in a prospective birth cohort study of Vietnamese infants. *J. Infect. Dis.* **200**, 1893–1900 (2009). doi:10.1086/648407 Medline

15. S. C. Kliks, S. Nimmanitya, A. Nisalak, D. S. Burke, Evidence that maternal dengue antibodies are important in the development of dengue hemorrhagic fever in infants. *Am. J. Trop. Med. Hyg.* **38**, 411–419 (1988). doi:10.4269/ajtmh.1988.38.411 Medline

16. H. Clapham, D. A. Cummings, A. Nisalak, S. Kalayanarooj, B. Thaisomboonsuk, C. Klungthong, S. Fernandez, A. Srikiatkhachorn, L. R. Macareo, J. Lessler, J. Reiser, I. K. Yoon, Epidemiology of infant dengue cases illuminates serotype-specificity in the interaction between immunity and disease, and changes in transmission dynamics. *PLOS Negl. Trop. Dis.* **9**, e0004262 (2015). doi:10.1371/journal.pntd.0004262 Medline

17. K. Laoprasopwattana, D. H. Libraty, T. P. Endy, A. Nisalak, S. Chunsuttiwat, D. W. Vaughn, G. Reed, F. A. Ennis, A. L. Rothman, S. Green, Dengue virus (DV) enhancing antibody activity in preillness plasma does not predict subsequent disease severity or viremia in secondary DV infection. *J. Infect. Dis.* **192**, 510–519 (2005). doi:10.1086/431520 Medline

18. D. H. Libraty, L. P. Acosta, V. Tallo, E. Segubre-Mercado, A. Bautista, J. A. Potts, R. G. Jarman, I. K. Yoon, R. V. Gibbons, J. D. Brion, R. Z. Capeding, A prospective nested case-control study of dengue in infants: Rethinking and refining the antibody-dependent enhancement dengue hemorrhagic fever model. *PLOS Med.* **6**, e1000171 (2009). doi:10.1371/journal.pmed.1000171 Medline

19. S. C. Kliks, A. Nisalak, W. E. Brandt, L. Wahl, D. S. Burke, Antibody-dependent enhancement of dengue virus growth in human monocytes as a risk factor for dengue hemorrhagic fever. *Am. J. Trop. Med. Hyg.* **40**, 444–451 (1989). doi:10.4269/ajtmh.1989.40.444 Medline

20. G. Kuan, A. Gordon, W. Avilés, O. Ortega, S. N. Hammond, D. Elizondo, A. Nuñez, J. Coloma, A. Balmaseda, E. Harris, The Nicaraguan Pediatric Dengue Cohort Study: Study design, methods, use of information technology, and extension to other infectious diseases. *Am. J. Epidemiol.* **170**, 120–129 (2009). doi:10.1093/aje/kwp092 Medline

21. See supplementary materials.

22. R. J. Fernández, S. Vázquez, Serological diagnosis of dengue by an ELISA inhibition method (EIM). *Mem. Inst. Oswaldo Cruz* **85**, 347–351 (1990). doi:10.1590/S0074-02761990000300012 Medline

23. R. de Alwis, K. L. Williams, M. A. Schmid, C. Y. Lai, B. Patel, S. A. Smith, J. E. Crowe, W. K. Wang, E. Harris, A. M. de Silva, Dengue viruses are enhanced by distinct populations of serotype cross-reactive antibodies in human immune sera. *PLOS Pathog.* **10**, e1004386 (2014). doi:10.1371/journal.ppat.1004386 Medline

24. A. Balmaseda, S. N. Hammond, Y. Tellez, L. Imhoff, Y. Rodriguez, S. I. Saborío, J. C. Mercado, L. Perez, E. Videa, E. Almanza, G. Kuan, M. Reyes, L. Saenz, J. J. Amador, E. Harris, High seroprevalence of antibodies against dengue virus in a prospective study of schoolchildren in Managua, Nicaragua. *Trop. Med. Int. Health* **11**, 935–942 (2006). doi:10.1111/j.1365-3156.2006.01641.x Medline

25. T. M. Therneau, A Package for Survival Analysis in S, version 2.38 (2015); https://CRAN.R-project.org/package=survival.

26. F. Narvaez, G. Gutierrez, M. A. Pérez, D. Elizondo, A. Nuñez, A. Balmaseda, E. Harris, Evaluation of the traditional and revised WHO classifications of dengue disease severity. *PLOS Negl. Trop. Dis.* **5**, e1397 (2011). doi:10.1371/journal.pntd.0001397 Medline

27. N. Alexander, A. Balmaseda, I. C. Coelho, E. Dimaano, T. T. Hien, N. T. Hung, T. Jänisch, A. Kroeger, L. C. Lum, E. Martinez, J. B. Siqueira, T. T. Thuy, I. Villalobos, E. Villegas, B. Wills; European Union, World Health Organization (WHO-TDR) supported DENCO Study Group, Multicentre prospective study on dengue classification in four South-east Asian and three Latin American countries. *Trop. Med. Int. Health* **16**, 936–948 (2011). doi:10.1111/j.1365-3156.2011.02793.x Medline

28. L. Meira-Machado, C. Cadarso-Suárez, F. Gude, A. Araújo, smoothHR: An R package for pointwise nonparametric estimation of hazard ratio curves of continuous predictors. *Comput. Math. Methods Med.* **2013**, 745742 (2013). doi:10.1155/2013/745742 Medline

29. L. C. Katzelnick, M. Montoya, L. Gresh, A. Balmaseda, E. Harris, Neutralizing antibody titers against dengue virus correlate with protection from symptomatic infection in a longitudinal cohort. *Proc. Natl. Acad. Sci. U.S.A.* **113**, 728–733 (2016). doi:10.1073/pnas.1522136113 Medline

30. D. Buddhari, J. Aldstadt, T. P. Endy, A. Srikiatkhachorn, B. Thaisomboonsuk, C. Klungthong, A. Nisalak, B. Khuntirat, R. G. Jarman, S. Fernandez, S. J. Thomas, T. W. Scott, A. L. Rothman, I. K. Yoon, Dengue virus neutralizing antibody levels associated with protection from infection in thai cluster studies. *PLOS Negl. Trop. Dis.* **8**, e3230 (2014). doi:10.1371/journal.pntd.0003230 Medline

31. S. A. Plotkin, P. B. Gilbert, Nomenclature for immune correlates of protection after vaccination. *Clin. Infect. Dis.* **54**, 1615–1617 (2012). doi:10.1093/cid/cis238 Medline

32. E. Simon-Lorière, V. Duong, A. Tawfik, S. Ung, S. Ly, I. Casadémont, M. Prot, N. Courtejoie, K. Bleakley, P. Buchy, A. Tarantola, P. Dussart, T. Cantaert, A. Sakuntabhai, Increased adaptive immune responses and proper feedback regulation protect against

clinical dengue. *Sci. Transl. Med.* **9**, eaal5088 (2017). doi:10.1126/scitranslmed.aal5088 Medline

33. L. C. Katzelnick, E. Harris; Participants in the Summit on Dengue Immune Correlates of Protection, Immune correlates of protection for dengue: State of the art and research agenda. *Vaccine* **35**, 4659–4669 (2017). doi:10.1016/j.vaccine.2017.07.045 Medline

34. A. Gordon, G. Kuan, J. C. Mercado, L. Gresh, W. Avilés, A. Balmaseda, E. Harris, The Nicaraguan Pediatric Dengue Cohort Study: Incidence of inapparent and symptomatic dengue virus infections, 2004-2010. *PLOS Negl. Trop. Dis.* **7**, e2462 (2013). doi:10.1371/journal.pntd.0002462 Medline

35. S. J. Thomas, A. Nisalak, K. B. Anderson, D. H. Libraty, S. Kalayanarooj, D. W. Vaughn, R. Putnak, R. V. Gibbons, R. Jarman, T. P. Endy, Dengue plaque reduction neutralization test (PRNT) in primary and secondary dengue virus infections: How alterations in assay conditions impact performance. *Am. J. Trop. Med. Hyg.* **81**, 825–833 (2009). doi:10.4269/ajtmh.2009.08-0625 Medline

36. Instituto de Medicina Tropical "Pedro Kourí," "Técnicas de laboratorio para el diagnóstico y la caracterización de los virus del dengue" (Pan American Health Organization, 2009).

37. WHO/TDR, "Dengue diagnostics: Proceedings of an international workshop" (WHO/TDR, 2004).

38. M. G. Guzman, S. B. Halstead, H. Artsob, P. Buchy, J. Farrar, D. J. Gubler, E. Hunsperger, A. Kroeger, H. S. Margolis, E. Martínez, M. B. Nathan, J. L. Pelegrino, C. Simmons, S. Yoksan, R. W. Peeling, Dengue: A continuing global threat. *Nat. Rev. Microbiol.* **8** (suppl.), S7–S16 (2010). doi:10.1038/nrmicro2460 Medline

39. S. Vázquez Ramudo, R. Fernández Llanes, *Rev. Cubana Med. Trop.* **41**, 18–26 (1989). Medline

40. E. Harris, E. Videa, L. Pérez, E. Sandoval, Y. Téllez, M. L. Pérez, R. Cuadra, J. Rocha, W. Idiaquez, R. E. Alonso, M. A. Delgado, L. A. Campo, F. Acevedo, A. Gonzalez, J. J. Amador, A. Balmaseda, Clinical, epidemiologic, and virologic features of dengue in the 1998 epidemic in Nicaragua. *Am. J. Trop. Med. Hyg.* **63**, 5–11 (2000). doi:10.4269/ajtmh.2000.63.5 Medline

41. A. Balmaseda, M. G. Guzmán, S. Hammond, G. Robleto, C. Flores, Y. Téllez, E. Videa, S. Saborio, L. Pérez, E. Sandoval, Y. Rodriguez, E. Harris, Diagnosis of dengue virus infection by detection of specific immunoglobulin M (IgM) and IgA antibodies in serum and saliva. *Clin. Diagn. Lab. Immunol.* **10**, 317–322 (2003). Medline

42. A. Balmaseda, K. Standish, J. C. Mercado, J. C. Matute, Y. Tellez, S. Saborío, S. N. Hammond, A. Nuñez, W. Avilés, M. R. Henn, E. C. Holmes, A. Gordon, J. Coloma, G. Kuan, E. Harris, Trends in patterns of dengue transmission over 4 years in a pediatric cohort study in Nicaragua. *J. Infect. Dis.* **201**, 5–14 (2010). doi:10.1086/648592 Medline

43. M. Montoya, L. Gresh, J. C. Mercado, K. L. Williams, M. J. Vargas, G. Gutierrez, G. Kuan, A. Gordon, A. Balmaseda, E. Harris, Symptomatic versus inapparent outcome in repeat dengue virus infections is influenced by the time interval between infections and study year. *PLOS Negl. Trop. Dis.* **7**, e2357 (2013). doi:10.1371/journal.pntd.0002357 Medline

44. A. Balmaseda, E. Sandoval, L. Pérez, C. M. Gutiérrez, E. Harris, Application of molecular typing techniques in the 1998 dengue epidemic in Nicaragua. *Am. J. Trop. Med. Hyg.* **61**, 893–897 (1999). doi:10.4269/ajtmh.1999.61.893 Medline

45. S. N. Hammond, A. Balmaseda, L. Pérez, Y. Tellez, S. I. Saborío, J. C. Mercado, E. Videa, Y. Rodriguez, M. A. Pérez, R. Cuadra, S. Solano, J. Rocha, W. Idiaquez, A. Gonzalez, E. Harris, Differences in dengue severity in infants, children, and adults in a 3-year hospital-based study in Nicaragua. *Am. J. Trop. Med. Hyg.* **73**, 1063–1070 (2005). Medline

46. A. Balmaseda, S. Saborio, Y. Tellez, J. C. Mercado, L. Pérez, S. N. Hammond, C. Rocha, G. Kuan, E. Harris, Evaluation of immunological markers in serum, filter-paper blood spots, and saliva for dengue diagnosis and epidemiological studies. *J. Clin. Virol.* **43**, 287–291 (2008). doi:10.1016/j.jcv.2008.07.016 Medline

47. C. Rocha, S. Silva, A. Gordon, S. N. Hammond, D. Elizondo, A. Balmaseda, E. Harris, Improvement in hospital indicators after changes in dengue case management in Nicaragua. *Am. J. Trop. Med. Hyg.* **81**, 287–292 (2009). Medline

48. K. Standish, G. Kuan, W. Avilés, A. Balmaseda, E. Harris, High dengue case capture rate in four years of a cohort study in Nicaragua compared to national surveillance data. *PLOS Negl. Trop. Dis.* **4**, e633 (2010). doi:10.1371/journal.pntd.0000633 Medline

49. M. Reyes, J. C. Mercado, K. Standish, J. C. Matute, O. Ortega, B. Moraga, W. Avilés, M. R. Henn, A. Balmaseda, G. Kuan, E. Harris, Index cluster study of dengue virus infection in Nicaragua. *Am. J. Trop. Med. Hyg.* **83**, 683–689 (2010). doi:10.4269/ajtmh.2010.10-0023 Medline

50. G. Gutierrez, K. Standish, F. Narvaez, M. A. Perez, S. Saborio, D. Elizondo, O. Ortega, A. Nuñez, G. Kuan, A. Balmaseda, E. Harris, Unusual dengue virus 3 epidemic in Nicaragua, 2009. *PLOS Negl. Trop. Dis.* **5**, e1394 (2011). doi:10.1371/journal.pntd.0001394 Medline

51. M. OhAinle, A. Balmaseda, A. R. Macalalad, Y. Tellez, M. C. Zody, S. Saborío, A. Nuñez, N. J. Lennon, B. W. Birren, A. Gordon, M. R. Henn, E. Harris, Dynamics of dengue disease severity determined by the interplay between viral genetics and serotype-specific immunity. *Sci. Transl. Med.* **3**, 114ra128 (2011). doi:10.1126/scitranslmed.3003084 Medline

52. A. Puschnik, L. Lau, E. A. Cromwell, A. Balmaseda, S. Zompi, E. Harris, Correlation between dengue-specific neutralizing antibodies and serum avidity in primary and secondary dengue virus 3 natural infections in humans. *PLOS Negl. Trop. Dis.* **7**, e2274 (2013). doi:10.1371/journal.pntd.0002274 Medline

53. P. Parameswaran, Y. Liu, K. M. Roskin, K. K. Jackson, V. P. Dixit, J. Y. Lee, K. L. Artiles, S. Zompi, M. J. Vargas, B. B. Simen, B. Hanczaruk, K. R. McGowan, M. A. Tariq, N. Pourmand, D. Koller, A. Balmaseda, S. D. Boyd, E. Harris, A. Z. Fire, Convergent antibody signatures in human dengue. *Cell Host Microbe* **13**, 691–700 (2013). doi:10.1016/j.chom.2013.05.008 Medline

54. S. J. Popper, A. Gordon, M. Liu, A. Balmaseda, E. Harris, D. A. Relman, Temporal dynamics of the transcriptional response to dengue virus infection in Nicaraguan

children. *PLOS Negl. Trop. Dis.* **6**, e1966 (2012). doi:10.1371/journal.pntd.0001966 Medline

55. C. Y. Lai, K. L. Williams, Y. C. Wu, S. Knight, A. Balmaseda, E. Harris, W. K. Wang, Analysis of cross-reactive antibodies recognizing the fusion loop of envelope protein and correlation with neutralizing antibody titers in Nicaraguan dengue cases. *PLOS Negl. Trop. Dis.* **7**, e2451 (2013). doi:10.1371/journal.pntd.0002451 Medline

56. L. C. Katzelnick, J. M. Fonville, G. D. Gromowski, J. Bustos Arriaga, A. Green, S. L. James, L. Lau, M. Montoya, C. Wang, L. A. VanBlargan, C. A. Russell, H. M. Thu, T. C. Pierson, P. Buchy, J. G. Aaskov, J. L. Muñoz-Jordán, N. Vasilakis, R. V. Gibbons, R. B. Tesh, A. D. M. E. Osterhaus, R. A. M. Fouchier, A. Durbin, C. P. Simmons, E. C. Holmes, E. Harris, S. S. Whitehead, D. J. Smith, Dengue viruses cluster antigenically but not as discrete serotypes. *Science* **349**, 1338–1343 (2015). doi:10.1126/science.aac5017 Medline

57. A. Balmaseda, K. Stettler, R. Medialdea-Carrera, D. Collado, X. Jin, J. V. Zambrana, S. Jaconi, E. Cameroni, S. Saborio, F. Rovida, E. Percivalle, S. Ijaz, S. Dicks, I. Ushiro-Lumb, L. Barzon, P. Siqueira, D. W. G. Brown, F. Baldanti, R. Tedder, M. Zambon, A. M. B. de Filippis, E. Harris, D. Corti, Antibody-based assay discriminates Zika virus infection from other flaviviruses. *Proc. Natl. Acad. Sci. U.S.A.* **114**, 8384–8389 (2017). doi:10.1073/pnas.1704984114 Medline

58. J. J. Waggoner, A. Balmaseda, L. Gresh, M. K. Sahoo, M. Montoya, C. Wang, J. Abeynayake, G. Kuan, B. A. Pinsky, E. Harris, Homotypic dengue virus reinfections in Nicaraguan children. *J. Infect. Dis.* **214**, 986–993 (2016). doi:10.1093/infdis/jiw099 Medline

59. J. J. Waggoner, L. Gresh, M. J. Vargas, G. Ballesteros, Y. Tellez, K. J. Soda, M. K. Sahoo, A. Nuñez, A. Balmaseda, E. Harris, B. A. Pinsky, Viremia and clinical presentation in Nicaraguan patients infected with Zika virus, chikungunya virus, and dengue virus. *Clin. Infect. Dis.* **63**, 1584–1590 (2016). doi:10.1093/cid/ciw589 Medline

60. S. Zompi, M. Montoya, M. O. Pohl, A. Balmaseda, E. Harris, Dominant cross-reactive B cell response during secondary acute dengue virus infection in humans. *PLOS Negl. Trop. Dis.* **6**, e1568 (2012). doi:10.1371/journal.pntd.0001568 Medline

61. T. P. Endy, K. B. Anderson, A. Nisalak, I. K. Yoon, S. Green, A. L. Rothman, S. J. Thomas, R. G. Jarman, D. H. Libraty, R. V. Gibbons, Determinants of inapparent and symptomatic dengue infection in a prospective study of primary school children in Kamphaeng Phet, Thailand. *PLOS Negl. Trop. Dis.* **5**, e975 (2011). doi:10.1371/journal.pntd.0000975 Medline

62. D. H. Clarke, J. Casals, Techniques for hemagglutination and hemagglutination-inhibition with arthropod-borne viruses. *Am. J. Trop. Med. Hyg.* **7**, 561–573 (1958). doi:10.4269/ajtmh.1958.7.561 Medline

63. R. S. Lanciotti, C. H. Calisher, D. J. Gubler, G. J. Chang, A. V. Vorndam, Rapid detection and typing of dengue viruses from clinical samples by using reverse transcriptase-polymerase chain reaction. *J. Clin. Microbiol.* **30**, 545–551 (1992). Medline

64. G. Kuno, I. Gómez, D. J. Gubler, Detecting artificial anti-dengue IgM immune complexes using an enzyme-linked immunosorbent assay.*Am. J. Trop. Med. Hyg.* **36**, 153–159 (1987). doi:10.4269/ajtmh.1987.36.153 Medline

65. M. Reed, A simple method of estimating fifty per cent endpoints. *Am. J. Hyg.* **27**, 493–497 (1938).

66. M. T. Alera, A. Srikiatkhachorn, J. M. Velasco, I. A. Tac-An, C. B. Lago, H. E. Clapham, S. Fernandez, J. W. Levy, B. Thaisomboonsuk, C. Klungthong, L. R. Macareo, A. Nisalak, L. Hermann, D. Villa, I. K. Yoon, Incidence of dengue virus infection in adults and children in a prospective longitudinal cohort in the Philippines. *PLOS Negl. Trop. Dis.* **10**, e0004337 (2016). doi:10.1371/journal.pntd.0004337 Medline

67. D. Leonard, leiv: Bivariate Linear Errors-In-Variables Estimation, R Package version 2.0-7 (2015).

68. A. C. Morrison, S. L. Minnick, C. Rocha, B. M. Forshey, S. T. Stoddard, A. Getis, D. A. Focks, K. L. Russell, J. G. Olson, P. J. Blair, D. M. Watts, M. Sihuincha, T. W. Scott, T. J. Kochel, Epidemiology of dengue virus in Iquitos, Peru 1999 to 2005: Interepidemic and epidemic patterns of transmission. *PLOS Negl. Trop. Dis.* **4**, e670 (2010). doi:10.1371/journal.pntd.0000670 Medline

69. R. C. Reiner Jr., S. T. Stoddard, B. M. Forshey, A. A. King, A. M. Ellis, A. L. Lloyd, K. C. Long, C. Rocha, S. Vilcarromero, H. Astete, I. Bazan, A. Lenhart, G. M. Vazquez-Prokopec, V. A. Paz-Soldan, P. J. McCall, U. Kitron, J. P. Elder, E. S. Halsey, A. C. Morrison, T. J. Kochel, T. W. Scott, Time-varying, serotype-specific force of infection of dengue virus. *Proc. Natl. Acad. Sci. U.S.A.* **111**, E2694–E2702 (2014). doi:10.1073/pnas.1314933111 Medline

70. M. Beltramello, K. L. Williams, C. P. Simmons, A. Macagno, L. Simonelli, N. T. Quyen, S. Sukupolvi-Petty, E. Navarro-Sanchez, P. R. Young, A. M. de Silva, F. A. Rey, L. Varani, S. S. Whitehead, M. S. Diamond, E. Harris, A. Lanzavecchia, F. Sallusto, The human immune response to dengue virus is dominated by highly cross-reactive antibodies endowed with neutralizing and enhancing activity. *Cell Host Microbe* **8**, 271–283 (2010). doi:10.1016/j.chom.2010.08.007 Medline

71. W. Dejnirattisai, W. Wongwiwat, S. Supasa, X. Zhang, X. Dai, A. Rouvinsky, A. Jumnainsong, C. Edwards, N. T. Quyen, T. Duangchinda, J. M. Grimes, W. Y. Tsai, C. Y. Lai, W. K. Wang, P. Malasit, J. Farrar, C. P. Simmons, Z. H. Zhou, F. A. Rey, J. Mongkolsapaya, G. R. Screaton, Corrigendum: A new class of highly potent, broadly neutralizing antibodies isolated from viremic patients infected with dengue virus. *Nat. Immunol.* **16**, 785 (2015). doi:10.1038/ni0715-785a Medline

72. W. Dejnirattisai, A. Jumnainsong, N. Onsirisakul, P. Fitton, S. Vasanawathana, W. Limpitikul, C. Puttikhunt, C. Edwards, T. Duangchinda, S. Supasa, K. Chawansuntati, P. Malasit, J. Mongkolsapaya, G. Screaton, Cross-reacting antibodies enhance dengue virus infection in humans. *Science* **328**, 745–748 (2010). doi:10.1126/science.1185181 Medline

73. S. A. Smith, Y. Zhou, N. P. Olivarez, A. H. Broadwater, A. M. de Silva, J. E. Crowe Jr., Persistence of circulating memory B cell clones with potential for dengue virus disease

enhancement for decades following infection. *J. Virol.* **86**, 2665–2675 (2012). doi:10.1128/JVI.06335-11 Medline

74. H. Loke, D. Bethell, C. X. Phuong, N. Day, N. White, J. Farrar, A. Hill, Susceptibility to dengue hemorrhagic fever in vietnam: Evidence of an association with variation in the vitamin d receptor and Fc gamma receptor IIa genes. *Am. J. Trop. Med. Hyg.* **67**, 102–106 (2002). doi:10.4269/ajtmh.2002.67.102 Medline

75. G. García, B. Sierra, A. B. Pérez, E. Aguirre, I. Rosado, N. Gonzalez, A. Izquierdo, M. Pupo, D. R. Danay Díaz, L. Sánchez, B. Marcheco, K. Hirayama, M. G. Guzmán, Asymptomatic dengue infection in a Cuban population confirms the protective role of the RR variant of the FcγRIIa polymorphism. *Am. J. Trop. Med. Hyg.* **82**, 1153–1156 (2010). doi:10.4269/ajtmh.2010.09-0353 Medline

76. S. N. Mohsin, S. Mahmood, A. Amar, F. Ghafoor, S. M. Raza, M. Saleem, Association of FcγRIIa polymorphism with clinical outcome of dengue infection: First insight from Pakistan. *Am. J. Trop. Med. Hyg.* **93**, 691–696 (2015). doi:10.4269/ajtmh.15-0199 Medline

77. M. K. Gentry, E. A. Henchal, J. M. McCown, W. E. Brandt, J. M. Dalrymple, Identification of distinct antigenic determinants on dengue-2 virus using monoclonal antibodies. *Am. J. Trop. Med. Hyg.* **31**, 548–555 (1982). doi:10.4269/ajtmh.1982.31.548 Medline

78. E. A. Henchal, M. K. Gentry, J. M. McCown, W. E. Brandt, Dengue virus-specific and flavivirus group determinants identified with monoclonal antibodies by indirect immunofluorescence. *Am. J. Trop. Med. Hyg.* **31**, 830–836 (1982). doi:10.4269/ajtmh.1982.31.830 Medline

79. G. Fibriansah, J. L. Tan, S. A. Smith, A. R. de Alwis, T. S. Ng, V. A. Kostyuchenko, K. D. Ibarra, J. Wang, E. Harris, A. de Silva, J. E. Crowe Jr., S. M. Lok, A potent anti-dengue human antibody preferentially recognizes the conformation of E protein monomers assembled on the virus surface. *EMBO Mol. Med.* **6**, 358–371 (2014). Medline

80. G. Fibriansah, K. D. Ibarra, T.-S. Ng, S. A. Smith, J. L. Tan, X.-N. Lim, J. S. G. Ooi, V. A. Kostyuchenko, J. Wang, A. M. de Silva, E. Harris, J. E. Crowe Jr., S.-M. Lok, Cryo-EM structure of an antibody that neutralizes dengue virus type 2 by locking E protein dimers. *Science* **349**, 88–91 (2015). doi:10.1126/science.aaa8651 Medline

81. G. Fibriansah, J. L. Tan, S. A. Smith, R. de Alwis, T. S. Ng, V. A. Kostyuchenko, R. S. Jadi, P. Kukkaro, A. M. de Silva, J. E. Crowe, S. M. Lok, A highly potent human antibody neutralizes dengue virus serotype 3 by binding across three surface proteins. *Nat. Commun.* **6**, 6341 (2015). doi:10.1038/ncomms7341 Medline

82. A. Rouvinski, P. Guardado-Calvo, G. Barba-Spaeth, S. Duquerroy, M. C. Vaney, C. M. Kikuti, M. E. Navarro Sanchez, W. Dejnirattisai, W. Wongwiwat, A. Haouz, C. Girard-Blanc, S. Petres, W. E. Shepard, P. Desprès, F. Arenzana-Seisdedos, P. Dussart, J. Mongkolsapaya, G. R. Screaton, F. A. Rey, Recognition determinants of broadly neutralizing human antibodies against dengue viruses. *Nature* **520**, 109–113 (2015). doi:10.1038/nature14130 Medline

83. S. A. Smith, A. R. de Alwis, N. Kose, E. Harris, K. D. Ibarra, K. M. Kahle, J. M. Pfaff, X. Xiang, B. J. Doranz, A. M. de Silva, S. K. Austin, S. Sukupolvi-Petty, M. S. Diamond, J.

E. Crowe Jr., The potent and broadly neutralizing human dengue virus-specific monoclonal antibody 1C19 reveals a unique cross-reactive epitope on the bc loop of domain II of the envelope protein. *mBio* **4**, e00873-13 (2013). Medline

84. S. Sukupolvi-Petty, S. K. Austin, M. Engle, J. D. Brien, K. A. Dowd, K. L. Williams, S. Johnson, R. Rico-Hesse, E. Harris, T. C. Pierson, D. H. Fremont, M. S. Diamond, Structure and function analysis of therapeutic monoclonal antibodies against dengue virus type 2. *J. Virol.* **84**, 9227–9239 (2010). doi:10.1128/JVI.01087-10 Medline

85. S. J. Balsitis, J. Coloma, G. Castro, A. Alava, D. Flores, J. H. McKerrow, P. R. Beatty, E. Harris, Tropism of dengue virus in mice and humans defined by viral nonstructural protein 3-specific immunostaining. *Am. J. Trop. Med. Hyg.* **80**, 416–424 (2009). Medline

86. D. Bates, M. Mächler, B. Bolker, S. Walker, Fitting linear mixed-effects models using lme4. *J. Stat. Softw.* **67**, 1–48 (2015). doi:10.18637/jss.v067.i01

87. J. E. Knowles, C. Frederick, merTools: Tools for Analyzing Mixed Effect Regression Models, R Package. version 0.3.0 (2016).

88. K. Desai, L. Coudeville, F. Bailleux, Modelling the long-term persistence of neutralizing antibody in adults after one dose of live attenuated Japanese encephalitis chimeric virus vaccine. *Vaccine* **30**, 2510–2515 (2012). doi:10.1016/j.vaccine.2012.02.005 Medline

89. N. Ferguson, R. Anderson, S. Gupta, The effect of antibody-dependent enhancement on the transmission dynamics and persistence of multiple-strain pathogens. *Proc. Natl. Acad. Sci. U.S.A.* **96**, 790–794 (1999). doi:10.1073/pnas.96.2.790 Medline

90. D. A. T. Cummings, I. B. Schwartz, L. Billings, L. B. Shaw, D. S. Burke, Dynamic effects of antibody-dependent enhancement on the fitness of viruses. *Proc. Natl. Acad. Sci. U.S.A.* **102**, 15259–15264 (2005). doi:10.1073/pnas.0507320102 Medline

91. N. G. Reich, S. Shrestha, A. A. King, P. Rohani, J. Lessler, S. Kalayanarooj, I.-K. Yoon, R. V. Gibbons, D. S. Burke, D. A. T. Cummings, Interactions between serotypes of dengue highlight epidemiological impact of cross-immunity. *J. R. Soc. Interface* **10**, 20130414 (2013). doi:10.1098/rsif.2013.0414 Medline

92. H. J. Wearing, P. Rohani, Ecological and immunological determinants of dengue epidemics. *Proc. Natl. Acad. Sci. U.S.A.* **103**, 11802–11807 (2006). doi:10.1073/pnas.0602960103 Medline

93. D. W. Vaughn, S. Green, S. Kalayanarooj, B. L. Innis, S. Nimmannitya, S. Suntayakorn, T. P. Endy, B. Raengsakulrach, A. L. Rothman, F. A. Ennis, A. Nisalak, Dengue viremia titer, antibody response pattern, and virus serotype correlate with disease severity. *J. Infect. Dis.* **181**, 2–9 (2000). doi:10.1086/315215 Medline

94. D. H. Libraty, P. R. Young, D. Pickering, T. P. Endy, S. Kalayanarooj, S. Green, D. W. Vaughn, A. Nisalak, F. A. Ennis, A. L. Rothman, High circulating levels of the dengue virus nonstructural protein NS1 early in dengue illness correlate with the development of dengue hemorrhagic fever. *J. Infect. Dis.* **186**, 1165–1168 (2002). doi:10.1086/343813 Medline

95. V. Tricou, N. N. Minh, J. Farrar, H. T. Tran, C. P. Simmons, Kinetics of viremia and NS1 antigenemia are shaped by immune status and virus serotype in adults with dengue. *PLOS Negl. Trop. Dis.* **5**, e1309 (2011). doi:10.1371/journal.pntd.0001309 Medline

96. H. E. Clapham, T. H. Quyen, D. T. Kien, I. Dorigatti, C. P. Simmons, N. M. Ferguson, Modelling virus and antibody dynamics during dengue virus infection suggests a role for antibody in virus clearance. *PLOS Comput. Biol.* **12**, e1004951 (2016). doi:10.1371/journal.pcbi.1004951 Medline

97. R. Ben-Shachar, S. Schmidler, K. Koelle, Drivers of inter-individual variation in dengue viral load dynamics. *PLOS Comput. Biol.* **12**, e1005194 (2016). doi:10.1371/journal.pcbi.1005194 Medline

98. M. N. Nguyet, T. H. Duong, V. T. Trung, T. H. Nguyen, C. N. B. Tran, V. T. Long, T. Dui, H. L. Nguyen, J. J. Farrar, E. C. Holmes, M. A. Rabaa, J. E. Bryant, T. T. Nguyen, H. T. C. Nguyen, L. T. H. Nguyen, M. P. Pham, H. T. Nguyen, T. T. H. Luong, B. Wills, C. V. V. Nguyen, M. Wolbers, C. P. Simmons, Host and viral features of human dengue cases shape the population of infected and infectious *Aedes aegypti* mosquitoes. *Proc. Natl. Acad. Sci. U.S.A.* **110**, 9072–9077 (2013). doi:10.1073/pnas.1303395110 Medline

99. V. Duong, L. Lambrechts, R. E. Paul, S. Ly, R. S. Lay, K. C. Long, R. Huy, A. Tarantola, T. W. Scott, A. Sakuntabhai, P. Buchy, Asymptomatic humans transmit dengue virus to mosquitoes. *Proc. Natl. Acad. Sci. U.S.A.* **112**, 14688–14693 (2015). doi:10.1073/pnas.1508114112 Medline

100. A. Handel, P. Rohani, Crossing the scale from within-host infection dynamics to between-host transmission fitness: A discussion of current assumptions and knowledge. *Philos. Trans. R. Soc. London Ser. B* **370**, 20140302 (2015). doi:10.1098/rstb.2014.0302 Medline

101. B. M. Althouse, K. A. Hanley, The tortoise or the hare? Impacts of within-host dynamics on transmission success of arthropod-borne viruses. *Philos. Trans. R. Soc. London Ser. B* **370**, 20140299 (2015). doi:10.1098/rstb.2014.0299 Medline

102. I. K. Yoon, A. L. Rothman, D. Tannitisupawong, A. Srikiatkhachorn, R. G. Jarman, J. Aldstadt, A. Nisalak, M. P. Mammen Jr., S. Thammapalo, S. Green, D. H. Libraty, R. V. Gibbons, A. Getis, T. Endy, J. W. Jones, C. J. Koenraadt, A. C. Morrison, T. Fansiri, C. Pimgate, T. W. Scott, Underrecognized mildly symptomatic viremic dengue virus infections in rural Thai schools and villages. *J. Infect. Dis.* **206**, 389–398 (2012). doi:10.1093/infdis/jis357 Medline

103. S. K. Austin, K. A. Dowd, B. Shrestha, C. A. Nelson, M. A. Edeling, S. Johnson, T. C. Pierson, M. S. Diamond, D. H. Fremont, Structural basis of differential neutralization of DENV-1 genotypes by an antibody that recognizes a cryptic epitope. *PLOS Pathog.* **8**, e1002930 (2012). doi:10.1371/journal.ppat.1002930 Medline
